# Cinnamil- and Quinoxaline-Derivative Indicator Dyes for Detecting Volatile Amines in Fish Spoilage

**DOI:** 10.3390/molecules24203673

**Published:** 2019-10-12

**Authors:** Xiaoyu Luo, Loong-Tak Lim

**Affiliations:** Department of Food Science, University of Guelph, Guelph, ON N1G 2W1, Canada

**Keywords:** cinnamil, quinoxaline, indicator dye, amine detection, intelligent packaging

## Abstract

Colorimetric indicators are versatile for applications such as intelligent packaging. By interacting with food, package headspace, and/or the ambient environment, color change in these indicators can be useful for reflecting the actual quality and/or monitoring distribution history (e.g., time and temperature) of food products. In this study, indicator dyes based on cinnamil and quinoxaline derivatives were synthesized using aroma compounds commonly present in food: diacetyl, benzaldehyde, *p*-tolualdehyde and *p*-anisaldehyde. The identities of cinnamil and quinoxaline derivatives were confirmed by Fourier transform infrared (FT–IR) spectroscopy, mass spectrometry (MS), ^1^H nuclear magnetic resonance (NMR) and ^13^C NMR analyses. Photophysical evaluation showed that the orange-colored cinnamil derivatives in dimethylsulfoxide (DMSO) turned to dark brownish coloration when exposed to strong alkalis. The cinnamil and acid-doped quinoxaline derivatives were sensitive to volatile amines commonly present during the spoilage in seafood. Quinoxaline derivatives doped by strong organic acid were effective as pH indicators for volatile amine detection, with lower detection limits than cinnamil. However, cinnamil exhibited more diverse color profiles than the quinoxaline indicators when exposed to ammonia, trimethylamine, triethylamine, dimethylamine, piperidine and hydrazine. Preliminary tests of acid-doped quinoxaline derivatives on fresh fish demonstrated their potential as freshness indicators in intelligent packaging applications.

## 1. Introduction

Packaging is an integral part of preservation system that is essential for food product distribution. Typical food packages provide physical protection against environmental factors such as light, oxygen, microbes, and so on [1]. On the other hand, intelligent packaging systems further incorporate one or more mechanisms to enhance the communication function of a package, such as integrating chemical sensing components to detect food spoilage and provide consumer feedback on actual product quality [2,3,4]. Reactive dyes and pigments, which exhibit changes in photophysical property upon the exposure to volatile compounds due to the spoilage from food, have been well-documented in the literature [2,3,5]. However, many such dyes are expensive and/or not suitable for food applications.

**Scheme 1:**
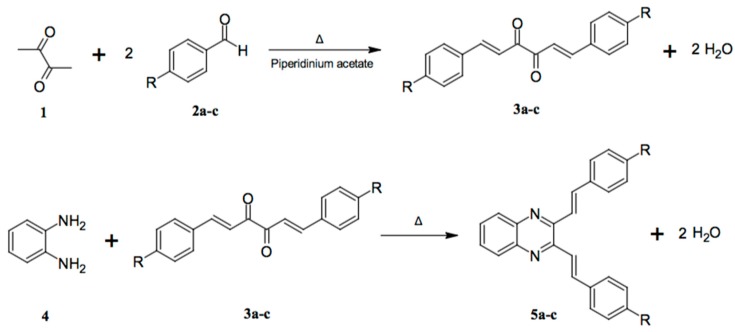
Synthesis route of cinnamil (**3a**–**c**) and quinoxaline (**5a**–**c**) derivatives, where **1** is diacetyl; **4** is *o*-phenylenediamine; R = H for compounds **2a**, **3a** and **5a**; R = CH_3_ for compounds **2b**, **3b** and **5b**; R = OCH_3_ for compounds **2c**, **3c** and **5c**.

Quinoxaline derivatives are colored and fluorescent materials due to their planar and highly conjugated structural configurations [6]. Apart from their photophysical properties, the quinoxaline ring, composed of a benzene ring and a pyrazine ring, is a key moiety that confers various bioactivities such as antibacterial, anti-inflammatory, anticancer and anti-malarial activities [7,8]. Researchers have proposed different approaches to synthesize quinoxaline derivatives, and the most common pathway is through the condensation reaction between 1,2-phenylenediamine and 1,2-dicarbonyl compounds [6,8,9,10], such as cinnamil, which is an orange-colored 1,2-diketone. Cinnamil can be synthesized from the condensation of 1:2 molar ratio diacetyl and benzaldehyde with piperidinium acetate as an alkaline catalyst [11]. This reaction follows the mechanism of Claisen–Schmidt condensation, where diacetyl is converted into an enolate that attacks the carbonyl group of benzaldehyde with subsequent water elimination [12]. Both substrates for cinnamil synthesis are commonly encountered in food. Diacetyl (**1** in Scheme 1; or 2,3-butanedione) is naturally occurring in butter, margarine, coffee, beer, and dairy products, as a buttery flavor ingredient [13,14,15]. On the other hand, benzaldehyde (**2a** in Scheme 1) is naturally present in almond, which is often serving as a food additive to mimic almond and cherry flavor [16,17]. Conceivably, cinnamil- and quinoxaline-derivative dyes of different photophysical properties can be synthetized by using other benzoic aldehydes, such as *p*-tolualdehyde from mango and kiwifruits [18,19], and *p*-anisaldehyde found in anise, vanilla and honey [20,21,22].

In this research, we synthesized cinnamil- and quinoxaline-derivative dyes via reacting diacetyl (**1** Scheme 1) with three benzoic aldehydes, namely benzaldehyde (**2a** Scheme 1), *p*-tolualdehyde (**2b** Scheme 1), and *p*-anisaldehyde (**2c** Scheme 1). The photophysical properties of the resulting dyes were characterized for the detection of volatile amines, such as ammonia, trimethylamine, triethylamine and dimethylamine, also known as total basic volatile nitrogen (TVBN). These amines are the main microbial degradation products of fish and shrimp during spoilage [23,24]. A preliminary study was conducted to illustrate the use of these dyes as colorimetric indicators for the freshness of fish.

## 2. Materials and Methods

### 2.1. Materials

Diacetyl, benzaldehyde, *p*-tolualdehyde, *p*-anisaldehyde, *o*-phenylenediamine, piperidine, *p*-toluenesulfonic acid (*p*-TsOH) and tetrabutylammonium hydroxide (TBAH) were purchased from Sigma-Aldrich, Oakville, ON, Canada. Methanol, dimethylsulfoxide (DMSO), glacial acetic acid, ammonium hydroxide, dimethylamine, trimethylamine, triethylamine, hydrazine and 250 mL French square bottles were purchased from Fisher Scientific, Mississauga, ON, Canada. Poly(ethylene terephthalate) (PET) plastic containers (with lids; 60 mL) were purchased from Reditainer, Guelph, ON, Canada.

### 2.2. Synthesis of Cinnamil and Quinoxaline Derivatives

Cinnamil derivatives (**3a**–**c**) were synthesized according to [11] with modifications. Benzoic aldehyde (0.4 mol) and diacetyl (0.1 mol) were added to 40 mL of methanol, followed by the addition of 2.0 mL of piperidine and 1.1 mL of glacial acetic acid as the catalyst. The mixture was heated at 95 °C under reflux for 2 h. The final solution was cooled in an ice bath for approximately 30 min. The crude products were obtained after vacuum filtration and solvent evaporation. The cinnamil derivatives were collected after recrystallization in methanol.

For quinoxaline (**5a**–**c**) synthesis, 4 mmol of cinnamil and 4 mmol of 1,2-phenylenediamine were added into a round bottom flask, followed by addition of 40 mL of methanol. The reaction mixture was heated immediately under reflux for 3–4 h, with constant stirring. The resulted solution was cooled, filtered, dried, and the crude product was recrystallized in methanol. The final products were collected after evaporating the solvent at 50 °C.

### 2.3. Instrumental Characterization

Infrared spectra of **3a**–**c** and **5a**–**c** in solids were recorded by Shimadzu IRPrestige-21 Fourier transform infrared (FT-IR) spectrophotometer (Shimadzu Corp., Kyoto, Japan), using the attenuated total reflectance (ATR) sampling technique, with 32 scans per sample in the mid-IR range (4000–600 cm^−1^). High-resolution liquid chromatography–mass spectrometry (LC-MS) was conducted on the synthesized dyes to confirm the molecular weights of the synthesized compounds. MS spectra were obtained by Agilent 6500 Series LC-MS system, with a quadruple-time of flight (Q-TOF) detector (Agilent Technologies, Santa Clara, CA, USA). ^1^H and ^13^C nuclear magnetic resonance (NMR) spectra were recorded by Bruker 400 MHz NMR spectrometer (Bruker Scientific Instruments, Billerica, MA, USA), using CDCl_3_ and DMSO-*d*_6_ as the solvent for cinnamil and quinoxaline derivatives respectively. Solvents for the synthesized indicator dyes were selected based on Hansen solubility parameter (HSP) concept, which estimates a material’s total cohesive energy by considering atomic dispersion, dipole-dipole interaction and hydrogen bonding interactions [25]. The solubility parameters of the synthesized compounds were calculated by the “DIY” function in the HSPiP software package (5.0.04, Hansen Solubility Parameters in Practice, www.hansen-solubility.com, Boca Raton, FL, USA), while the HSP values of DMSO and CHCl_3_ were acquired from the software’s library.

Ultraviolet-visible (UV-Vis) absorbance (280–700 nm, with 1.0 nm interval) of the synthesized dyes in DMSO (10 µM) was measured by Thermo Scientific Evolution 60S UV–Visible spectrophotometer (Thermo Electron Scientific Instruments LLC, Madison, WI, USA), at room temperature.

### 2.4. Detection of Volatile Amines

For the detection of volatile amines, a piece of cellulose paper (2.5 × 2.5 cm^2^) was wetted in either 100 µL acid doped quinoxaline (**5a**–**c**) solution (1:5000 quinoxaline:*p*-TsOH) or 100 µL 20 mM cinnamil (**3a**). After drying, the indicator-impregnated papers were attached to the inner wall of 250 mL French square glass bottles. Each of the volatile amines (7.0 mmol) was added to the bottle and the color responses (L*, a* and b* values) before and after 1 h exposure at room temperature were determined by Konica Minolta CR-300 chroma meter (Konica Minolta Sensing Americas, NJ, USA). Amines were added in excess amount to ensure the saturation of dye indicators was achieved. Each treatment was performed in triplicates.

The overall color change (ΔE) was calculated by:

(1) $$\Delta E\;=\;\sqrt{{\left(\Delta L^{*}\right)}^{2}+{\left(\Delta a^{*}\right)}^{2}+{\left(\Delta b^{*}\right)}^{2}}$$

where ΔL* is the change of lightness, Δa* is the change of redness or greenness, and Δb* is the change of yellowness or blueness.

Acid-doped quinoxaline (**5a**–**c**) and cinnamil (**3a**) were exposed to different concentrations of volatile amines to determine their limit of detection (LOD) and limit of quantitation (LOQ) values. Based on the calibration curve of the color response (ΔE) versus concentration (μg/mL), LOD and LOQ were determined by:

LOD = 3.3 σ/S(2)

LOQ = 10 σ/S(3)

where σ is the root-mean-square deviation and S is the slope of the fitted line of the calibration curve.

### 2.5. Preliminary Evaluation on Fish Freshness Indication

Preliminary evaluation of the indicator for the detection of fish spoilage was conducted by using the acid-doped quinoxaline compound **5b**. A piece of cellulose paper was dosed with 100 μL acid-doped quinoxaline **5b** (1:5000 quinoxaline:*p*-TsOH), dried, and attached to the inside of the PET containers. Four types of fresh fish fillets were purchased from a local grocery store (Market Fresh, Guelph, Canada): haddock, perch, cod and tilapia. For each replicate, approximately 20 g of fish sample or 20 mL of water (control), was added to the PET container and stored at room temperature. Color change of the indicator was monitored by capturing images at different storage time.

### 2.6. Data Analysis

FT-IR spectra were analyzed by IR Solution software (Shimadzu Corp., Kyoto, Japan). All NMR spectra were processed and analyzed by TopSpin software 4.0.3 (Bruker Scientific Instruments, Billerica, MA, USA). For discriminating color responses of different amine treatments, analysis of variance (ANOVA) on ΔL*, Δa*, Δb* and ΔΕ values followed by Tukey honest significant difference (HSD), with 95% confidence interval, were conducted by R software 3.3.3 (R Foundation for Statistical Computing, Vienna, Austria).

## 3. Results and Discussion

Physical appearance, % yield, NMR, IR and MS data for each of the synthesized indicator dyes are summarized below:

*(1E,5E)-1,6-diphenyl-1,5-hexadiene-3,4-dione (**3a**)* Orange crystal; 10.20% yield; ^1^H NMR (400 MHz, CDCl_3_) δ = 7.87 (d, *J* = 16.2 Hz, 2H), 7.66 (dd, *J* = 7.7, 1.9 Hz, 4H), 7.48 (d, *J* = 16.2 Hz, 2H), 7.46–7.40 (m, 6H) ppm; ^13^C NMR (101 MHz, CDCl_3_) δ = 189.22, 148.00, 134.56, 131.55, 129.20, 129.14, 119.78 ppm; IR (neat) ν_max_ = 1668, 1591, 1572, 1447, 986, 752, 719, 687 cm^−1^; mass (electrospray ionization (ESI) LC-MS): 263.1045 (M + 1).

*(1E,5E)-1,6-bis(4-methylphenyl)-1,5-hexadiene-3,4-dione (**3b**)* Orange crystal; 8.51% yield; ^1^H NMR (400 MHz, CDCl_3_) δ = 7.84 (d, *J* = 16.1 Hz, 2H), 7.55 (d, *J* = 8.0 Hz, 4H), 7.42 (d, *J* = 16.1 Hz, 2H), 7.23 (d, *J* = 8.0 Hz, 4H), 2.40 (s, 6H) ppm; ^13^C NMR (101 MHz, CDCl_3_) δ = 189.57, 148.01, 142.25, 131.89, 129.93, 129.16, 118.95, 21.76 ppm; IR (neat) ν_max_ = 1668, 1593, 1510, 1327, 1182, 995, 800, 683 cm^−1^; mass (ESI LC-MS): 291.1361 (M + 1).

*(1E,5E)-1,6-bis(4-methoxyphenyl)-1,5-hexadiene-3,4-dione (**3c**)* Orange crystal; 9.43% yield; ^1^H NMR (400 MHz, CDCl_3_) δ = 7.82 (d, *J* = 15.9 Hz, 2H), 7.61 (d, *J* = 9.0 Hz, 4H), 7.33 (d, *J* = 15.9 Hz, 2H), 6.94 (d, *J* = 9.0 Hz, 4H), 3.86 (s, 6H) ppm; ^13^C NMR (101 MHz, CDCl_3_) δ = 189.69, 162.48, 147.67, 131.03, 127.43, 117.74, 114.66, 55.59 ppm; IR (neat) ν_max_ = 1678, 1593, 1506, 1417, 1248, 1175, 1028, 991, 829, 812, 787, 687 cm^−1^; mass (ESI LC-MS): 323.1273 (M + 1).

*2,3-Bis[(E)-2-phenylvinyl]-quinoxaline (**5a**)* Yellow powder; 82.71% yield; ^1^H NMR (400 MHz, DMSO-*d*_6_) δ = 8.09 (d, *J* = 15.2 Hz, 2H), 8.06–8.04 (m, 2H), 8.01 (d, *J* = 15.2 Hz, 2H), 7.94 (d, *J* = 7.4 Hz, 4H), 7.80-7.78 (m, 2H), 7.48 (t, *J* = 7.4 Hz, 4H), 7.41 (t, *J* = 7.4 Hz, 2H) ppm; ^13^C NMR (101 MHz, DMSO-*d*_6_) δ = 148.67, 141.00, 137.37, 136.11, 129.91, 129.16, 128.82, 128.59, 128.07, 122.46 ppm; IR (neat) ν_max_ = 1624, 1518, 1495, 1194, 1130, 964, 745, 688 cm^−1^; mass (ESI LC-MS): 335.1512 (M + 1).

*2,3-Bis[(E)-2-(4-methylphenyl)vinyl]-quinoxaline (**5b**)* Yellow powder; 84.84% yield; ^1^H NMR (400 MHz, DMSO-*d*_6_) δ = 8.04-8.00 (m, 4H), 7.97 (d, *J* = 15.6 Hz, 2H), 7.83 (d, *J* = 8.2 Hz, 4H), 7.78-7.75 (m, 2H), 7.29 (d, *J* = 8.2 Hz, 4H), 2.37 (s, 6H) ppm; ^13^C NMR (101 MHz, DMSO-*d*_6_) δ = 148.77, 140.93, 138.87, 137.34, 133.42, 129.71, 129.42, 128.52, 128.06, 121.36, 21.02 ppm; IR (neat) ν_max_ = 1622, 1510, 1190, 1128, 966, 799, 752 cm^−1^; mass (ESI LC-MS): 363.1850 (M + 1).

*2,3-Bis[(E)-2-(4-methoxyphenyl)vinyl]-quinoxaline (**5c**)* Yellow powder; 75.70% yield; ^1^H NMR (400 MHz, DMSO-*d*_6_) δ = 8.01–7.99 (m, 2H), 7.99–7.92 (m, 4H), 7.88 (d, *J* = 8.7 Hz, 4H), 7.75–7.73 (m, 2H), 7.03 (d, *J* = 8.8 Hz, 4H), 3.83 (s, 6H) ppm; ^13^C NMR (101 MHz, DMSO-*d*_6_) δ = 160.17, 148.90, 140.86, 137.01, 129.67, 129.48, 128.90, 128.43, 119.99, 114.27, 55.27 ppm; IR (neat) ν_max_ = 1620, 1597, 1508, 1419, 1248, 1173, 1022, 970, 830, 810, 762 cm^−1^; mass (ESI LC-MS): 395.1746 (M + 1).

The yields for the cinnamil derivatives (**3a**–**c**) were between 8.51 to 10.20%, similar to those reported in the literature [11]. The low yield can be explained on the basis of the intrinsic chemical properties of the substrates. Since diacetyl is a Brønsted acid, this reaction is initiated via the enolization of diacetyl’s carbonyl group under alkaline conditions provided by piperidinium acetate. In theory, the addition reaction between one mole of enolized diacetyl and two moles of benzoic aldehydes (**2a**–**c**) should result in one mole of **3a**–**c**. However, in this alkaline solution, the non-enolizable benzoic aldehydes would undergo a self-redox reaction known as Cannizzaro reaction, generating equal molar amount of benzoic alcohol and benzoic acid [26]. Moreover, self-condensation of diacetyl could occur as another side reaction. Preliminary experiments with piperidinium acetate gave the highest yield over other catalysts tested, including sodium acetate, sodium hydroxide, and sodium citrate. Hence, piperidinium acetate was selected as the base in this reaction. Considering the low costs of the reaction substrates and the readily achievable experimental protocol, the overall 10% yield is probably a fair compromise. In contrast, the synthesis of quinoxaline derivatives (**5a**–**c**) via 1,2-diamine and 1,2-diketone condensation had higher yields ranging from 75–84%, similar to that reported in a previous study involving the same reaction [10].

### 3.1. Structural Confirmation

The molecular structures of the synthesized cinnamil and quinoxaline derivatives were confirmed by MS, FT-IR and NMR spectroscopies. The molecular weights of **3a**–**c** and **5a**–**c** were confirmed from MS results. In FT-IR spectroscopy, the carbonyl groups of cinnamil derivatives (**3a**–**c**) were shown as strong IR stretching bands at 1678–1668 cm^−1^. As compared to **3a** and **3b**, the carbonyl band of **3c** shifted to higher frequency due to the presence of the ether group. The C-O stretching band of **3c** appeared at 1028 cm^−1^. For **3a**–**c**, absorbance bands between 1600 and 1400 cm^−1^ could be attributed to C=C stretching of *trans* double bonds and aromatic rings. The strong IR absorbance around 990 cm^−1^ was due to the *trans* vinyl C=C-H out-of-plane bending. The substitution in the benzene ring could be identified by the aromatic C-H out-of-plane bending vibration at 800 cm^−1^ for **3b** and **3c** (*para*-di-substitution) and two strong bands at 752 and 719 cm^−1^ for **3a** (mono-substitution). In ^1^H NMR, the *trans* carbon–carbon double bond can be identified as doublets with a large *^3^J* coupling constant of approximately 16 Hz. All aromatic proton peaks appeared around 7–8 ppm. The methyl group proton peaks for **3b** and **3c** appeared as a singlet, at a lower chemical shift due to more diamagnetic shielding as compared to **3a**. In ^13^C NMR, the carbonyl carbon peak was located at high chemical shift (189 ppm) due to strong electronegativity effect, while the methyl group carbons for **3b** and **3c** were at the lower end (21 ppm and 55 ppm, respectively).

For quinoxaline derivatives (**5a**–**c**), the imine (C=N) stretching band was located at 1624–1620 cm^−1^ in the IR spectra. Similar to **3a**–**c**, the C=C stretching band for **5a**–**c** appeared around 1600–1400 cm^−1^ and the *trans* C=C-H out-of-plane bending at 970–964 cm^−1^. The ether group of **5c** can be identified by the C-O stretching band at 1022 cm^−1^. In ^1^H NMR spectra, the *trans* C=C bond were confirmed by the two doublets with a coupling constant of 15–16 Hz. The aromatic protons on the quinoxaline backbone appeared as multiplets, while the doublets with lower coupling (7–9 Hz) could be attributed to other aromatic protons. The two singlet peaks of **5b** and **5c** (at 2.37 ppm and 3.83 ppm, respectively) confirmed the conservation of the methyl groups. In ^13^C NMR spectra, the carbonyl carbon peaks of **5b** and **5c** disappeared, while the methyl groups appeared, at similar chemical shifts as in **3b** and **3c**. For **5c**, the peak of the quaternary aromatic carbon next to the ether oxygen was observed at 160 ppm. Overall, the IR and NMR results corroborated well with those from previous research [10].

### 3.2. Photophysical Properties

**Figure 1:**
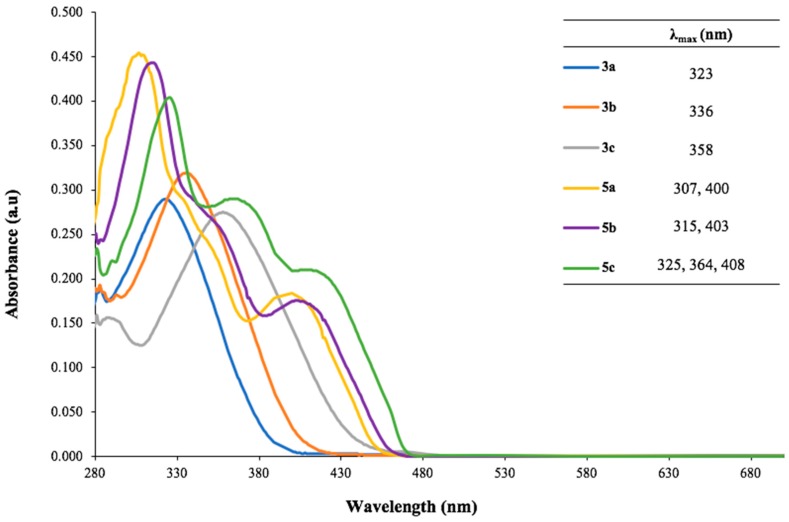
Ultraviolet (UV)-visible absorbance spectra (280–700 nm) of 10 μM cinnamil derivatives (**3a**–**c**) and quinoxaline derivatives (**5a**–**c**) in dimethylsulfoxide (DMSO).

The synthesized cinnamil (**3a**–**c**) and quinoxaline (**5a**–**c**) derivatives appeared as orange and pale-yellow solids, respectively. Figure 1 shows the absorption spectra in UV-Vis wavelength, along with the maximum absorbance wavelength (λ_max_) values, of the dyes dissolved in DMSO. The highly conjugated moiety of all synthesized compounds induced a bathochromic shift, resulting in absorption of the visible light. The absorption peaks of **3a**–**c** are mainly attributed to π-π* electronic transition of the carbonyl group. The UV-Vis spectra of quinoxaline derivatives **5a**–**c** have two or more maxima, due to the electronic transitions of π-π* (305–325 nm) and n-π* (400–410 nm) of C=N bonds within the pyrazine ring. Moreover, **5c** showed a maximum at 364 nm, suggesting the n-σ* transition of C-O bond, which was absent in **5a** and **5b**. Substitution of the aromatic rings with electron-donating groups resulted in red shifts for both **3a**–**c** and **5a**–**c**. These findings are consistent with those reported in the literature [10].

**Figure 2:**
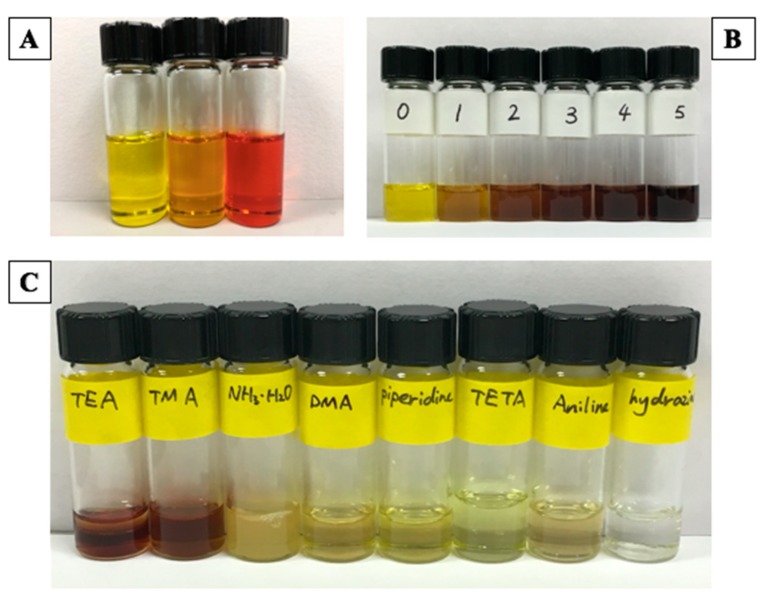
(**A**) Color of quinoxaline derivatives **5a**, **5b**, and **5c** (from left to right; 10 µM in DMSO) doped with *p*-TsOH (5000:1 acid:dye molar ratio). (**B**) Color of 20 µM cinnamil **3a** in DMSO with the addition of tetrabutylammonium hydroxide (TBAH): 0 = no TBAH, 1 = 0.3 µmol TBAH, 2 = 0.6 µmol TBAH, 3 = 0.9 µmol TBAH, 4 = 1.2 µmol TBAH, 5 = 1.5 µmol TBAH. (**C**) Color of 20 µM cinnamil **3a** in DMSO with the addition of 0.1 mmol different amines (from left to right). TEA: triethylamine; TMA: trimethylamine; ammonium hydroxide; DMA: dimethylamine; piperidine; TETA: triethylenetetramine, aniline, and hydrazine.

The photophysical properties of cinnamil and quinoxaline derivatives were examined by reacting with acids and amines in aqueous solutions. Figure 2A illustrated the color appearance of quinoxaline derivatives with excess amount of *p*-TsOH as a dopant. The dopant was added at 5000:1 acid:dye molar ratio to completely protonate the nitrogen atoms within the pyrazine ring of **5a**–**c**. The colors of acid-doped quinoxaline solutions were strikingly different as shown in Figure 2A, changing to bright yellow, orange and reddish orange for **5a**, **5b**, and **5c** respectively. This color variation can be explained by the bathochromic shift of **5a** to **5c**, as shown in the absorbance spectra (Figure 1). The pH values of 10 µM **5a**, **5b** and **5c** with *p*-TsOH in DMSO were determined to be 1.87, 1.82 and 1.62 respectively. The UV-Vis spectra of acid-doped **5a**–**c** were shown in the supplementary file (Figure S25).

Cinnamil (**3a**), being a 1,2-diketone with α-hydrogens, enolized under strong alkaline conditions, underwent yellow-to-dark brown transition when doped with TBAH (Figure 2B). Specifically, the α-hydrogens can be deprotonated in the presence of a strong base, resulting in negatively charged α-carbons, lowering the energy gap of π-π* transition, and causing bathochromic shift. Increasing TBAH concentration raised the alkalinity of the solution, inducing the bathochromic shift and resulting in a darker solution. The photophysical property of cinnamil was further evaluated with ammonium hydroxide and other amines in aqueous solutions, including, tertiary amines, secondary amines and primary amines (Figure 2C). Ammonium hydroxide and tertiary amines trimethylamine (TMA) and triethylamine (TEA) showed similar color effects as TBAH, due to their strong alkalinity and lacking nucleophilic properties of the protonated nitrogen. The increasing color intensity observed with NH_3_, TMA and TEA was attributed to their different pKa values, which are 9.25, 9.80 and 10.81, respectively at room temperature [27,28]. Under these alkaline conditions, cinnamil’s diketone moiety would be converted to the corresponding enolate form. For secondary amines such as dimethylamine and piperidine, cinnamil solution remained yellow after 4 h (Figure 2C). This phenomenon could be due to the excess secondary amine present, which served as a nucleophile that attacked the carbonyl centre of cinnamil, forming an unstable aminoalcohol structure that tended to convert back to cinnamil at equilibrium, giving the solution’s bright yellow color. In comparison, the cinnamil solution exhibited a reduced color intensity when doped with more reactive primary amines (e.g., triethylenetetramine (TETA), aniline, and hydrazine) by forming imine groups through ketone-amine condensation. On the other hand, the yellow color of cinnamil disappeared by reacting with hydrazine, which introduced an additional amino substitute to each imine group (i.e., hydrazone), leading to a further hypsochromic shift. In summary, cinnamil displayed different color responses to various classes of amines, suggesting that it is potentially useful for amine discrimination.

### 3.3. Volatile Amine Detection

#### 3.3.1. Amine Detection by Quinoxaline Derivatives

**Figure 3:**
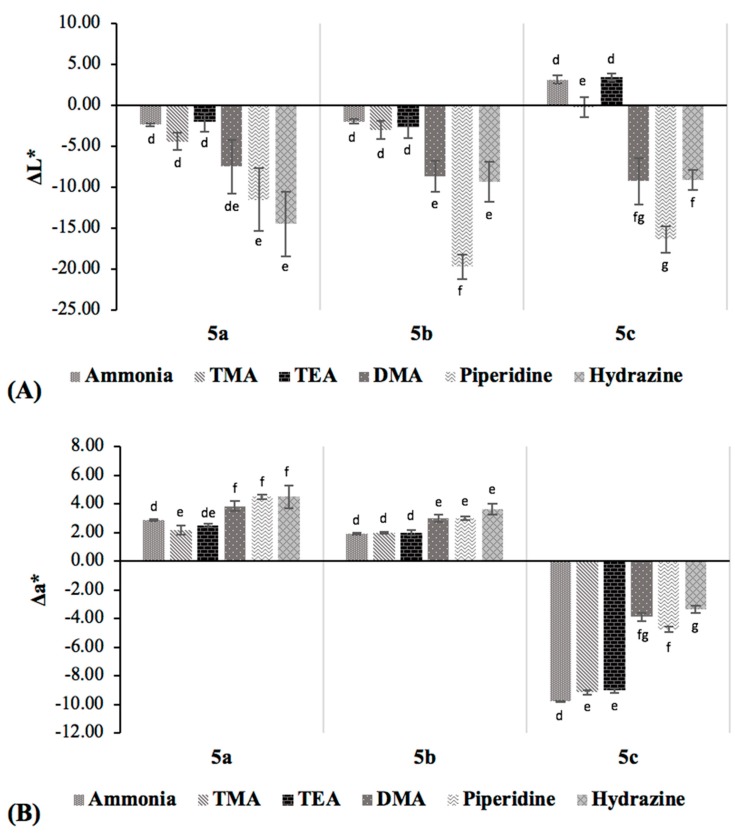

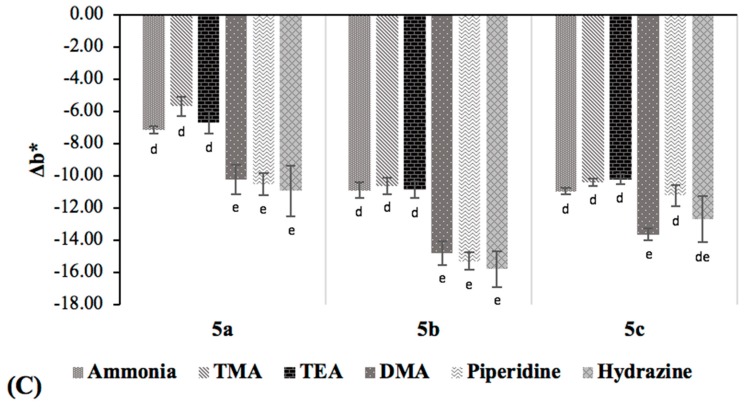
Changes of L* (**A**), a* (**B**), and b* (**C**) of acid-doped quinoxalines **5a**–**c** before and after the exposure to amines. Columns labelled with different letters represent significant changes in color parameters for each of the indicator dyes (*p* < 0.05). TMA: trimethylamine; TEA: triethylamine; DMA: dimethylamine.

The acid-doped quinoxaline derivative solutions, as shown in Figure 2A, were used as colorimetric indicators for ammonia, trimethylamine, dimethylamine, triethylamine, piperidine and hydrazine. The relative basicity with different pKa values of ammonia, TMA and TEA have been discussed in Section 3.2. By doping the quinoxaline derivatives **5a**–**c** with *p*-TsOH (Figure 2A), they were protonated and effectively functioning as pH indicators when exposed to these alkaline volatiles. The changes in color profiles for these acid-doped quinoxaline derivatives are shown in Figure 3.

The different initial colors observed among the acid-doped quinoxaline solutions were due to the different aromatic substituents (Figure 2A). In general, the color of the indicators became darker upon amine exposure, as indicated by the decreasing L* value, except **5c**. Quinoxaline derivative **5c** showed a considerable decrease in a* value, consistent with the fading of its orange reddish color when exposed to the alkalis. On the other hand, small increases in a* value were detected for **5a** and **5b**. All samples exhibited decreases in b* values (i.e., decreases in yellow color), the extent of which was less for tertiary amines (TMA and TEA) and ammonia, as compared to the primary and secondary amines. However, the color variations among **5a**, **5b** and **5c** were not readily distinguished by the naked eye, implying that the quinoxaline compounds would not allow the discrimination of the six alkaline volatiles. Hence, these dyes are more useful for the detection of the volatile amine mixtures, such as those produced during the spoilage of fresh fish and fishery products.

#### 3.3.2. Amine Detection by Cinnamil

**Figure 4:**
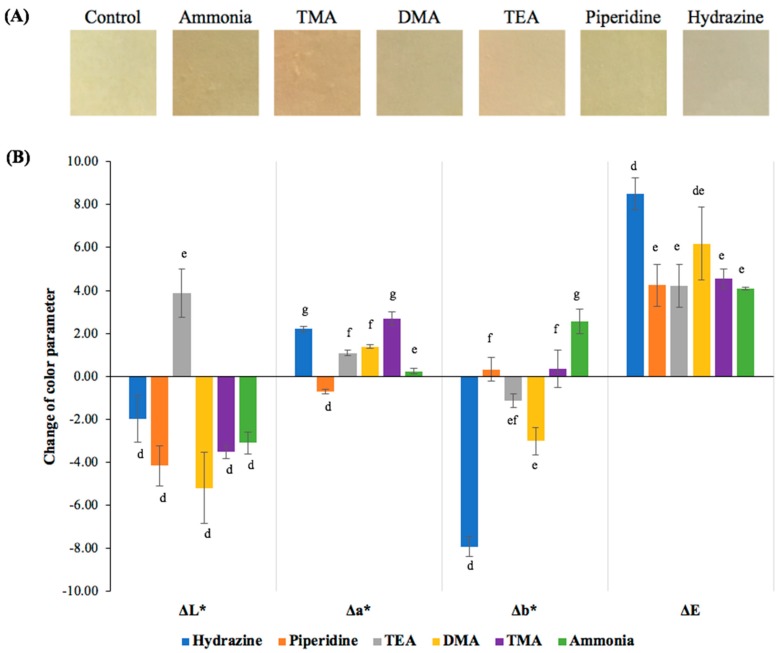
(**A**) Representative color responses of cinnamil-loaded 2 × 2 cm^2^ cellulose papers when exposed to different volatile amines. Sample labelled as “control” depicts color without amine addition. (**B**) Changes in color parameters (L*, a*, b* and ΔΕ) after the exposure to different amines. TEA: triethylamine; DMA: dimethylamine; and TMA: trimethylamine. Columns of the same color parameter with different lowercase letters are significantly different (*p* < 0.05).

The color response images of cinnamil-loaded cellulose papers to different amines are shown in Figure 4A, and the changes of individual color parameters are summarized in Figure 4B. Figure 4B shows that ΔΕ value for hydrazine was significantly higher than those of the other four amines tested (i.e., piperidine, TEA, TMA and ammonia, except for dimethylamine (DMA)), although other amines could not be differentiated by relying on the ΔE values alone. However, L* value increased when cinnamil was exposed to TEA, while all other amines showed decreasing trends. This observation implies that L* value can potentially be considered for differentiating TEA from other amines. Similarly, the a* value declined when cinnamil was exposed to piperidine, but increased for all the other amines, especially hydrazine and TMA. On the other hand, minimal change in a* value was observed for ammonia, which can be used to differentiate it from other volatiles. Hydrazine resulted in a significant decrease in b*, consistent with the visual decrease in yellow color (Figure 4A), while insignificant changes in b* values were observed for piperidine and TMA. Taken together, by investigating the L*, a* and b* color parameters individually, all six amines tested can be differentiated by cinnamil. These results demonstrate that cinnamil can potentially be used as a colorimetric indicator for the discrimination of volatile amines. To stabilize the dye and prevent leaching into food product, encapsulating it within a protective matrix permeable to the amine analytes is important for real food packaging applications.

#### 3.3.3. Limit of Detection (LOD) and Limit of Quantitation (LOQ) for Amine Detection

The LOD and LOQ values of **3a** and acid-doped **5a**–**c** for detecting different volatile amines are summarized in Table 1, showing that the detection and quantitation limits of cinnamil were higher than the acid-doped quinoxaline compounds. The lower sensitivity of **3a** than quinoxaline compounds is consistent with findings in Figure 3 and Figure 4, i.e., when exposing to the same concentration of a given amine, the extent of color changes for **3a** was lower than **5a**–**c**. Comparing the acid-doped quinoxaline dyes, the LOD and LOQ values were the lowest (i.e., most sensitive) for **5c** when exposed to low concentrations of amines. Among the six amines, ammonia resulted in the lowest LOD and LOQ values for all indicators, while triethylamine the highest. Conceivably, the detection limit can be further improved by optimizing the concentration of the indicator dyes, the amount of acid dopant, and/or changing the substrates for dye deposition. Since trimethylamine, ammonia and dimethylamine were the predominant volatile compounds produced during the spoilage of fish, the low detection limits of these dyes for these compounds are promising for intelligent packaging applications to monitor fish freshness.

### 3.4. Preliminary Test on Fish Freshness Indication by Acid-Doped Quinoxaline

During the spoilage of fish, the predominate volatile amine formed is TMA, due to microbial activities from the bacteria such as *Shewanella putrifaciens*, *Photobacterium phosphoreum*, and *Vibrionacaea* spp. [29,30]. These bacteria derive energy by converting trimethylamineoxide (TMAO), an osmoregulatory compound in fish tissue, to TMA that further breaks down to DMA [31]. Apart from TMA and DMA, ammonia can also be generated during fish spoilage from the proliferation of aerobic bacteria [29]. In view of the prevalence of these basic nitrogen volatiles during the spoilage of fish, a preliminary study was conducted to illustrate the application of an acid-doped quinoxaline derivative, **5b**, as freshness indicator.

**Figure 5:**
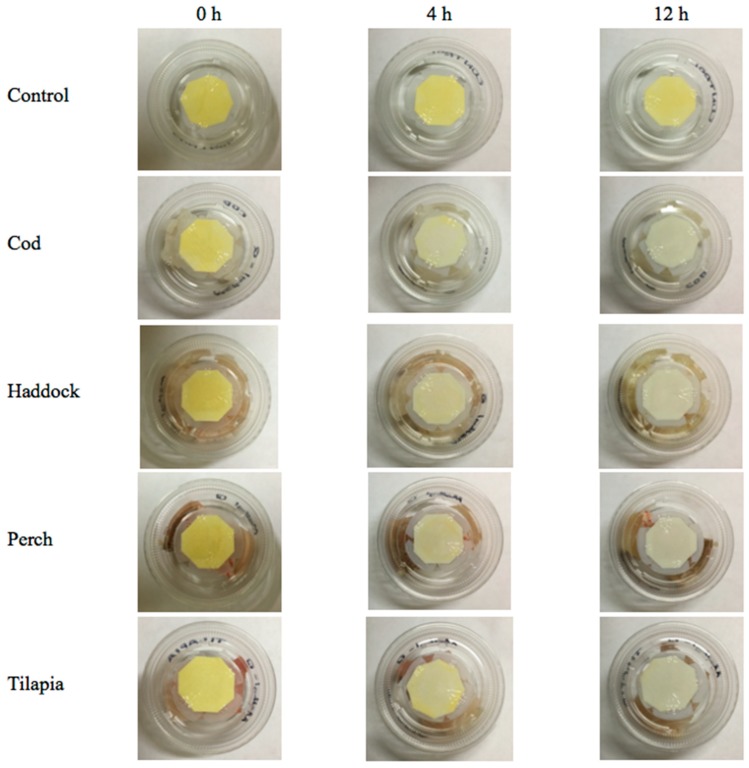
Color response images captured for using acid-doped quinoxaline **5b**-loaded cellulose paper as an indicator to test different fish fillets: cod, haddock, perch, and tilapia, under accelerated condition at room temperature over a 12 h period, where “Control” denotes the container was filled with water with similar weight of the fish fillets in other treatments.

The color responses of the **5b** indicators, prepared as described in Section 2.5, are shown in Figure 5. It can be seen that the **5b**-containing cellulose paper was in bright yellowish color at the beginning. The yellow color faded notably after 4-h of exposure to various fish fillets stored at room temperature, and substantially faded after 12 h. This observation correlated well with the results presented in Figure 3, where b* value decreased dramatically when the acid-doped quinoxaline was exposed to volatile amines. The reduction in yellowness intensity was attributable to the interaction of basic amines present in the package headspace with the dopant, *p*-TsOH. Accordingly, the extent of color change will be affected by the quantity of acid dopant in the dye; the higher the dopant concentration, the lower the indicator’s sensitivity towards the basic volatiles, and vice versa. This preliminary study shows that the acid-doped quinoxaline dyes are promising as colorimetric indicators for real-time detection of fish freshness. More future studies are needed to correlate the color profile of the dyes with the concentration of total volatile basic nitrogen compounds, as well as the quality parameters of the fish fillets (e.g., microbial loads, texture profiles, sensory properties), in order to accurately establish the color–freshness relationship.

## 4. Conclusions

In this study, cinnamil and quinoxaline dyes were synthesized from substrates commonly found in food. The molecular structures of the synthesized dyes were confirmed by FT-IR and NMR spectroscopies. Quinoxaline derivatives in solutions showed color variations with different aromatic substituents. Cinnamil in aqueous solution showed different color responses to different amine compounds. Tertiary amines enolized the carbonyl groups of cinnamil and darkened the solution. Secondary and primary amines reacted with cinnamil to generate aminoalcohol and imine groups respectively. Acid-doped quinoxaline derivatives were employed for detecting different basic amines: ammonia, trimethylamine, dimethylamine, triethylamine, piperidine and hydrazine. The results dictated significant color changes after 1 h exposure, but these pH indicators were unable to differentiate between the different amines tested. In a preliminary study, an acid-doped quinoxaline compound (**5b**) showed significant color changes with time when exposed to the headspace of test PET packages containing different fish fillets. Cinnamil displayed significant changes of color parameters, suggesting that it is also potentially useful as a colorimetric indicator for amine discrimination. In comparison, the acid-doped quinoxaline **5a**–**c** showed a higher sensitivity with lower LOD and LOQ values, as compared to cinnamil. Among the six different amines being tested, ammonia, trimethylamine and dimethylamine showed lower LOD and LOQ values than other amine compounds.

Future studies to investigate the dyes’ kinetics of color change, at different analyte concentrations, will be useful to construct predictive mathematical models. Methods need to be developed to incorporate the dye into actual package system, such as via standalone label/sticker, printing on package substrate, dispersion into film structure, and so on. To this end, the stability of the dyes during storage and end-use conditions must be evaluated to ensure end-use efficacy. In terms of safety, a strategy is needed to prevent the migration of the dye to food, especially in direct contact application, by exploiting semi-permeable protective coating/film. Finally, cytotoxicity of the dyes will need to be evaluated to gain legislative approval for use in commercial food products. Besides potential use in intelligent packaging of food, the synthesized dyes may find uses in other areas such as pharmacy and environmental sciences for volatile amine detection.

## Figures and Tables

**Table 1:** Limit of detection (LOD) and limit of quantitation (LOQ) (µg/mL) of using **3a** and acid-doped **5a**–**c** as the colorimetric indicators for the detection of different volatile amines.

Amine	3a		Acid-Doped 5a		Acid-Doped 5b		Acid-Doped 5c	
	LOD	LOQ	LOD	LOQ	LOD	LOQ	LOD	LOQ
TMA	332.5	1007.5	311.0	942.3	200.7	608.2	119.6	362.4
DMA	114.9	348.2	173.5	525.9	53.8	162.9	56.6	171.5
TEA	1544.4	4680.0	504.4	1528.3	626.7	1899.2	386.9	1172.6
Ammonia	43.5	144.9	32.9	99.7	31.3	94.8	19.8	60.1
Piperidine	945.1	2864.0	871.5	2640.8	446.7	1353.5	452.9	1372.6
Hydrazine	348.0	1054.5	488.4	1480.0	380.0	1151.5	291.3	882.9

TMA: trimethylamine; DMA: dimethylamine; TEA: triethylamine.

## Supplementary Materials

The following are available online at https://www.mdpi.com/1420-3049/24/20/3673/s1, Figures S1–S12: ^1^H and ^13^C NMR spectra of synthesized indicator compounds **3a**–**c** and **5a**–**c**; Figures S13–S18: FT-IR spectra of synthesized indicator compounds **3a**–**c** and **5a**–**c**; Figures S19–S24: MS spectra of synthesized indicator compounds **3a**–**c** and **5a**–**c**; Figure S25: UV-Vis spectra of acid-doped quinoxaline derivatives **5a**–**c**.

## References

[1] Pereira de Abreu D.A., Cruz J.M., Paseiro Losada P. (2012). Active and intelligent packaging for the food industry. Food Rev. Int..

[2] Ghaani M., Cozzolino C.A., Castelli G., Farris S. (2016). An overview of the intelligent packaging technologies in the food sector. Trends Food Sci. Technol..

[3] Lim L.T., Yada R. (2011). Active and intelligent packaging materials. Comprehensive Biotechnology.

[4] Yam K.L., Takhistov P.T., Miltz J. (2005). Intelligent packaging: Concepts and applications. J. Food Sci..

[5] Kerry J.P., O’Grady M.N., Hogan S.A. (2006). Past, current and potential utilisation of active and intelligent packaging systems for meat and muscle-based products: A review. Meat Sci..

[6] Jaung J.-Y. (2006). Synthesis and halochromism of new quinoxaline fluorescent dyes. Dye. Pigment..

[7] Pereira J.A., Pessoa A.M., Cordeiro M.N.D.S., Fernandes R., Prudêncio C., Noronha J.P., Vieira M. (2015). Quinoxaline, its derivatives and applications: A state of the art review. Eur. J. Med. Chem..

[8] Tariq S., Somakala K., Amir M. (2018). Quinoxaline: An insight into the recent pharmacological advances. Eur. J. Med. Chem..

[9] Guo W.-X., Jin H.-L., Chen J.-X., Chen F., Ding J.-C., Wu H.-Y. (2009). An efficient catalyst-free protocol for the synthesis of quinoxaline derivative under ultrasound irradiation. J. Braz. Chem. Soc..

[10] Thirumurugan P., Muralidharan D., Perumal P.T. (2009). The synthesis and photophysical studies of quinoxaline and pyridopyrazine derivatives. Dye. Pigment..

[11] Constable E.C., Hannon M.J., Smith D.R. (1994). Cinnamil—An oilgopridine precursor. Tetrahedron Lett..

[12] Kim D.H., Rahman A.F.M.M., Jeong B.-S., Lee E.S., Jahng Y. (2009). Acetate-promoted aldol-type reaction: Scope and reactivity of acetates and aldehydes. Bull. Korean Chem. Soc..

[13] Clark S., Winter C.K. (2015). Diacetyl in foods: A review of safety and sensory characteristics. Compr. Rev. Food Sci. Food Saf..

[14] Pires E.J., Teixeira J.A., Brányik T., Brandão T., Vicente A.A. (2015). Continuous beer fermentation—Diacetyl as a villain. J. Inst. Brew..

[15] Sanz C., Maeztu L., Zapelena M.J., Bello J., Cid C. (2002). Profiles of volatile compounds and sensory analysis of three blends of coffee: Influence of different proportions of Arabica and Robusta and influence of roasting coffee with sugar. J. Sci. Food Agric..

[16] Loch C., Reusch H., Ruge I., Godelmann R., Pflaum T., Kuballa T., Schumacher S., Lachenmeier D.W. (2016). Benzaldehyde in cherry flavour as a precursor of benzene formation in beverages. Food Chem..

[17] Verma R.S., Padalia R.C., Singh V.R., Goswami P., Chauhan A., Bhukya B. (2017). Natural benzaldehyde from *Prunus persica* (L.) Batsch. Int. J. Food Prop..

[18] Pino J.A., Queris O. (2011). Analysis of volatile compounds of mango wine. Food Chem..

[19] Jordán M.J., Margaría C.A., Shaw P.E., Goodner K.L. (2002). Aroma active components in aqueous kiwi fruit essence and kiwi fruit puree by GC-MS and multidimensional GC/GCO. J. Agric. Food Chem..

[20] Chen X., Zhang X., Meng R., Zhao X., Zhao Z., Liu Z., Shi C., Guo N. (2016). Efficacy of a combination of nisin and *p*-anisaldehyde against *Listeria monocytogenes*. Food Control.

[21] Takahashi M., Inai Y., Miyazawa N., Kurobayashi Y., Fujita A. (2013). Identification of the key odorants in Tahitian cured vanilla beans (*Vanilla tahitensis*) by GC-MS and an aroma extract dilution analysis. Biosci. Biotechnol. Biochem..

[22] Yang Y., Battesti M.-J., Costa J., Dupuy N., Paolini J. (2018). Volatile components as chemical markers of the botanical origin of Corisan honeys. Flavour Fragr. J..

[23] Kuswandi B., Jayus, Oktaviana R., Abdullah A., Heng L.Y. (2014). A novel on-package sticker sensor based on methyl red for real-time monitoring of broiler chicken cut freshness. Packag. Technol. Sci..

[24] Morsy M.K., Zór K., Kostesha N., Alstrøm T.S., Heiskanen A., El-Tanahi H., Sharoba A., Papkovsky D., Larsen J., Khalaf H. (2016). Development and validation of a colorimetric sensor array for fish spoilage monitoring. Food Control.

[25] Hansen C.M. (2007). Hansen Solubility Parameters: A User’s Handbook.

[26] Arora B., Pandey P.S., Gupta M.N. (2014). Lipase catalyzed Cannizzaro-type reaction with substituted benzaldehydes in water. Tetrahedron Lett..

[27] Burckhardt B.C., Thelen P. (1995). Effect of primary, secondary and tertiary amines on membrane potential and intracellular pH in *Xenopus laevis* oocytes. Pflug. Arch..

[28] Konopacka-Łyskawa D., Kościelska B., Karczewski J., Gołąbiewska A. (2017). The influence of ammonia and selected amines on the characteristics of calcium carbonate precipitated from calcium chloride solutions via carbonation. Mater. Chem. Phys..

[29] Church N. (1998). MAP fish and crustacean—Sensory enhancement. Food Sci. Technol. Today.

[30] Ghaly A.E., Dave D., Budge S., Brooks M.S. (2010). Fish spoilage mechanisms and preservation techniques: Review. Am. J. Appl. Sci..

[31] Gram L., Dalgaard P. (2002). Fish spoilage bacteria—Problems and solutions. Curr. Opin. Biotechnol..

