# Supplementary material for: Cinnamil- and Quinoxaline-Derivative Indicator Dyes for Detecting Volatile Amines in Fish Spoilage
Source: Molecules. 2019 Oct 12;24(20):3673. doi: 10.3390/molecules24203673 (PMC6832431; doi:10.3390/molecules24203673)
Supplement: Supplementary file 1 [file molecules-24-03673-s001.pdf]

Supporting Information:

Cinnamil and quinoxaline derivative indicator dyes for detecting volatile amines in fish spoilage

Xiaoyu LUO, Loong-Tak LIM *

Department of Food Science, University of Guelph, Guelph, ON Canada, N1G 2W1

Table S1. Structures of each synthesized indicator dyes

Dye	Structure	Substitution for R groups
Cinnamil Derivatives	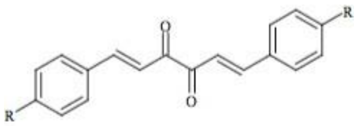 **3a-c**	**3a**: R=H<br>**3b**: R=CH_3<br>**3c**: R=OCH_3
Quinoxaline Derivatives	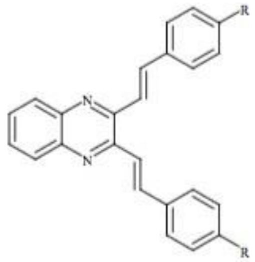 **5a-c**	**5a**: R=H<br>**5b**: R=CH_3<br>**5c**: R=OCH_3

* Corresponding author. Email: llim@uoguelph.ca, Tel: 1-519-824-4120 ext. 56586

Table of Contents

	Page
**Figure S1.** ^{1}H NMR spectrum of cinnamil **3a** in $CDCl_3$	3
**Figure S2.** ^{13}C NMR JMOD spectrum of cinnamil **3a** in $CDCl_3$	4
**Figure S3.** ^{1}H NMR spectrum of cinnamil derivative **3b** in $CDCl_3$	5
**Figure S4.** ^{13}C NMR JMOD spectrum of cinnamil derivative **3b** in $CDCl_3$	6
**Figure S5.** ^{1}H NMR spectrum of cinnamil derivative **3c** in $CDCl_3$	7
**Figure S6.** ^{13}C NMR JMOD spectrum of cinnamil derivative **3c** in $CDCl_3$	8
**Figure S7.** ^{1}H NMR spectrum of quinoxaline derivative **5a** in DMSO-d_6	9
**Figure S8.** ^{13}C NMR JMOD spectrum of quinoxaline derivative **5a** in DMSO-d_6	10
**Figure S9.** ^{1}H NMR spectrum of quinoxaline derivative **5b** in DMSO-d_6	11
**Figure S10.** ^{13}C NMR JMOD spectrum of quinoxaline derivative **5b** in DMSO-d_6	12
**Figure S11.** ^{1}H NMR spectrum of quinoxaline derivative **5c** in DMSO-d_6	13
**Figure S12.** ^{13}C NMR JMOD spectrum of quinoxaline derivative **5c** in DMSO-d_6	14
**Figure S13.** ATR-FTIR absorbance spectrum of cinnamil **3a**	15
**Figure S14.** ATR-FTIR absorbance spectrum of cinnamil derivative **3b**	16
**Figure S15.** ATR-FTIR absorbance spectrum of cinnamil derivative **3c**	17
**Figure S16.** ATR-FTIR absorbance spectrum of quinoxaline derivative **5a**	18
**Figure S17.** ATR-FTIR absorbance spectrum of quinoxaline derivative **5b**	19
**Figure S18.** ATR-FTIR absorbance spectrum of quinoxaline derivative **5c**	20
**Figure S19.** MS spectrum of cinnamil **3a**	21
**Figure S20.** MS spectrum of cinnamil derivative **3b**	22
**Figure S21.** MS spectrum of cinnamil derivative **3c**	23
**Figure S22.** MS spectrum of quinoxaline derivative **5a**	24
**Figure S23.** MS spectrum of quinoxaline derivative **5b**	25
**Figure S24.** MS spectrum of quinoxaline derivative **5c**	26
**Figure S25.** UV-Vis absorbance spectra of (1:5000 quinoxaline:*p*TsOH molar ratio) acid-doped quinoxaline derivatives **5a-c** in DMSO	27

1H NMR (400 MHz)
Compound 3a in CDCl3

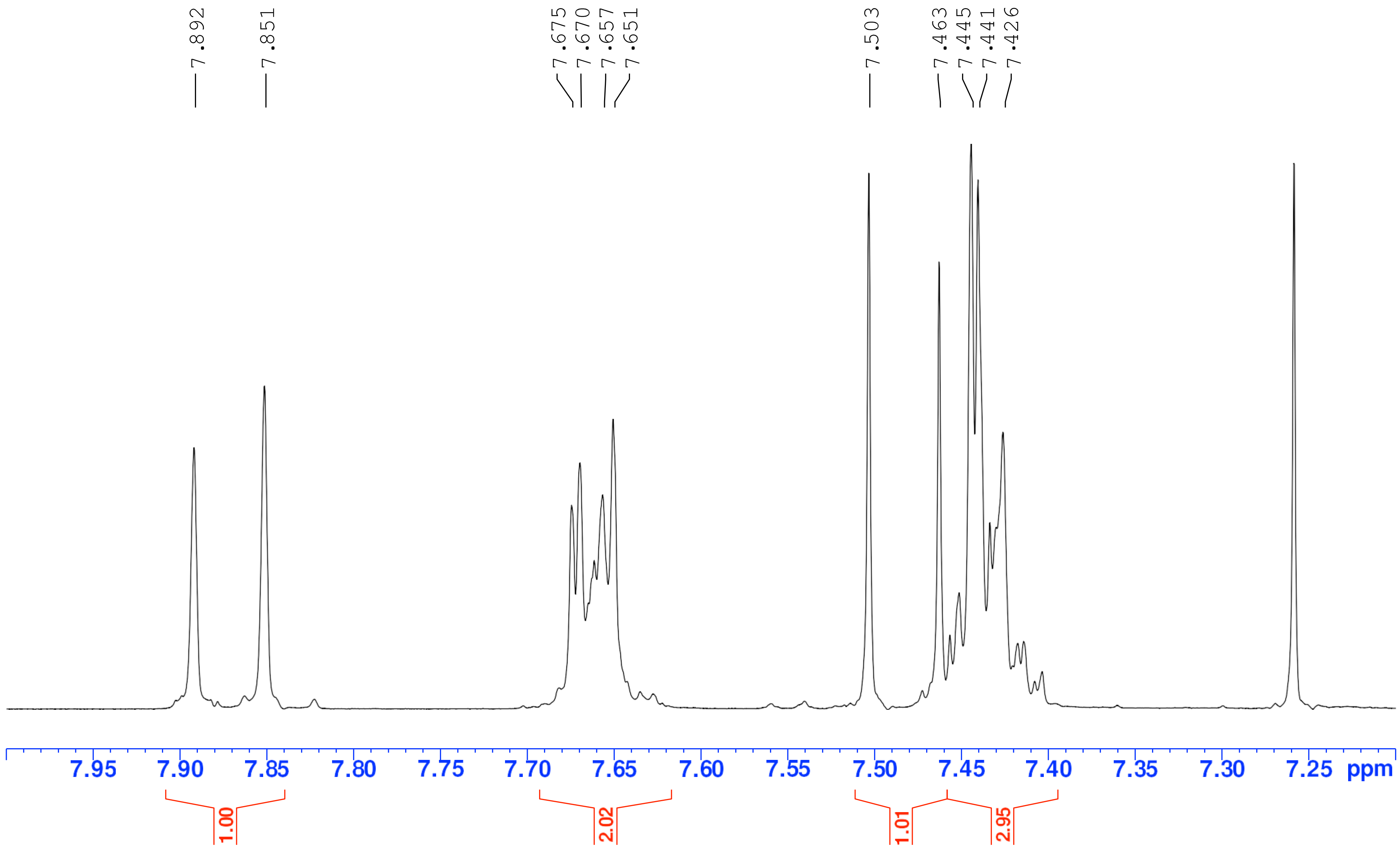

13C JMOD NMR (101 MHz)
Quaternary and CH2 up, CH3 and CH down
Compound 3a in CDCl3

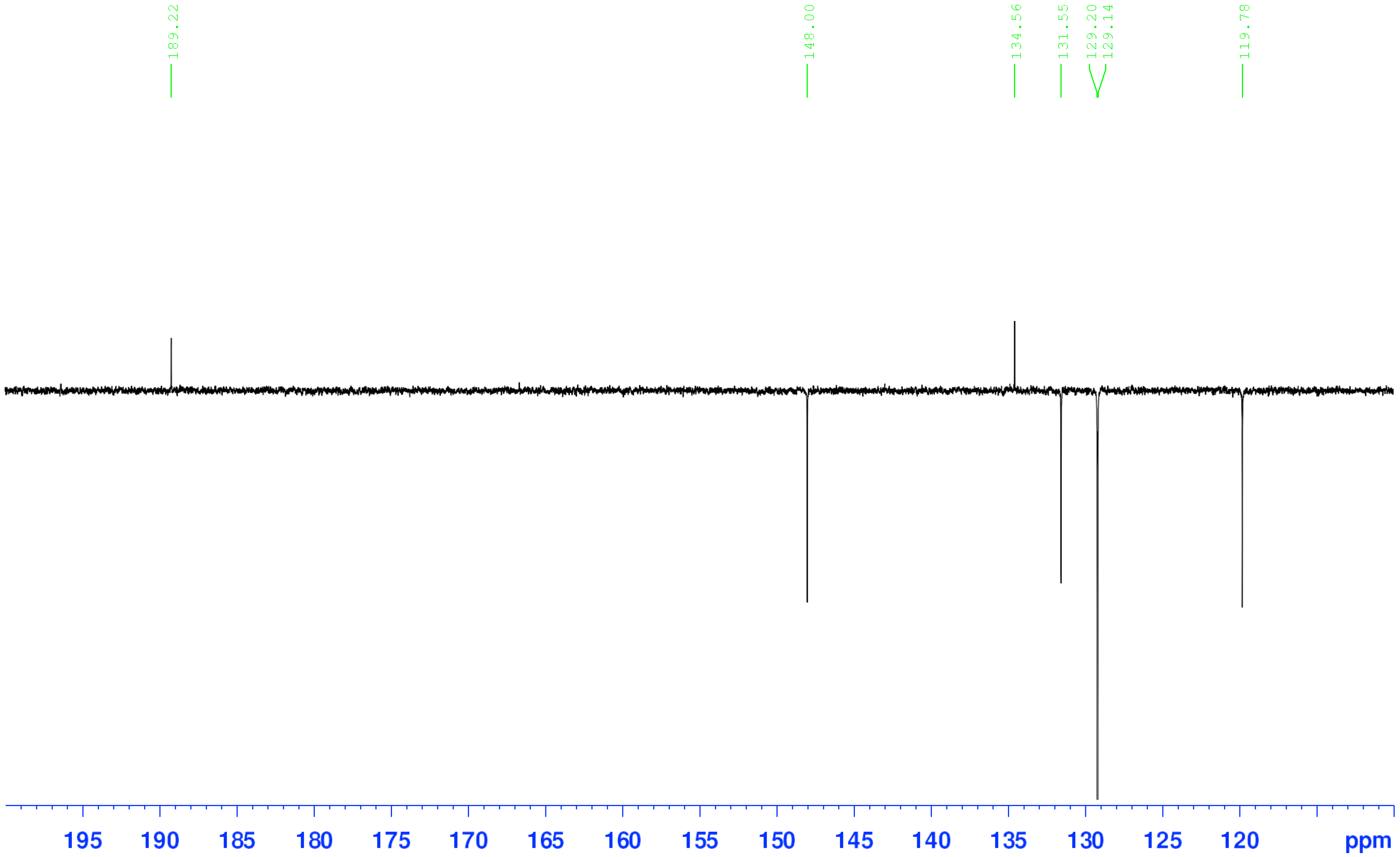

1H NMR (400 MHz)
Compound 3b in CDCl3

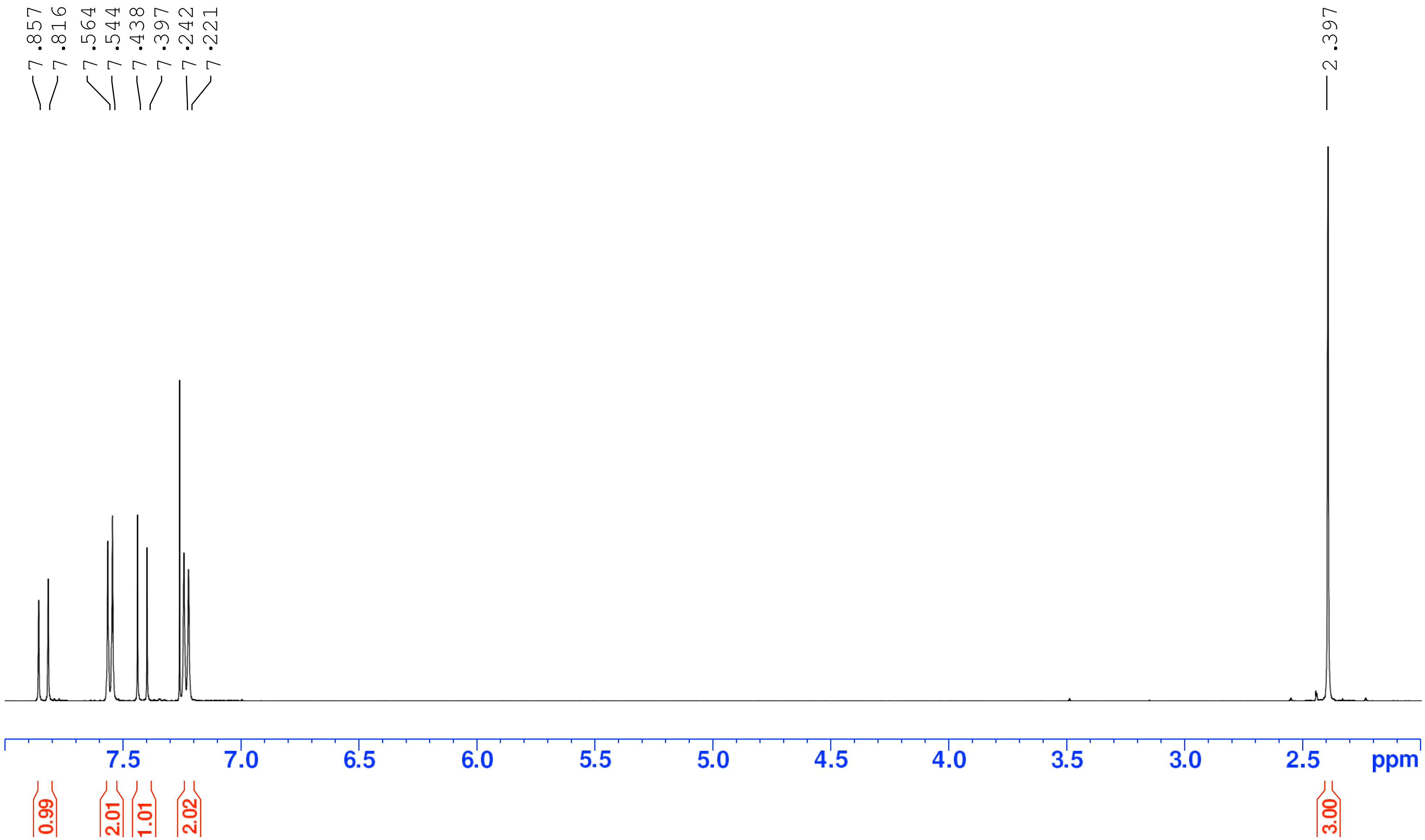

13C JMOD NMR (101 MHz)
Quaternary and CH2 up, CH3 and CH down
Compound 3b in CDCl3

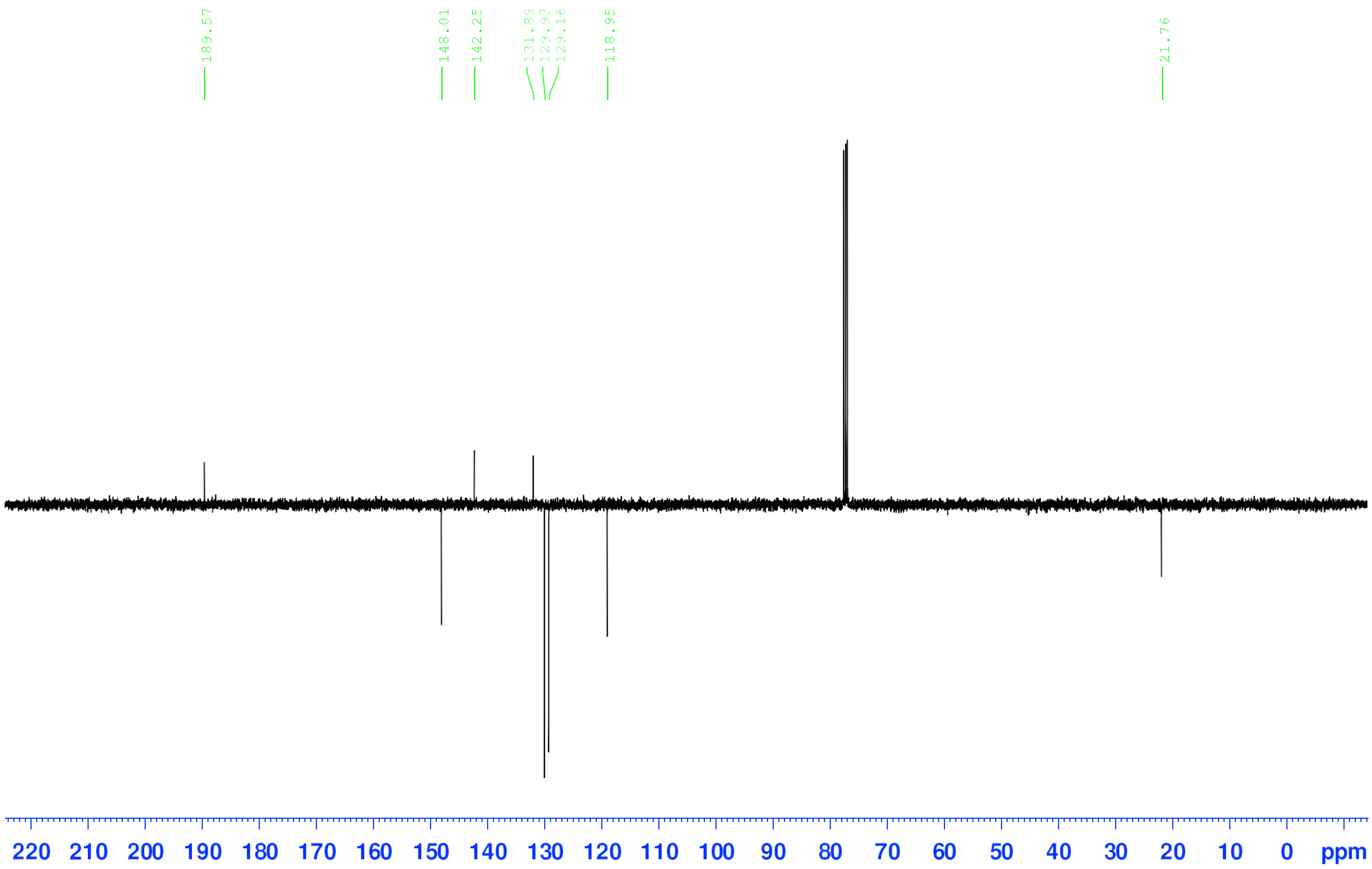

1H NMR (400 MHz)
Compound 3c in CDCl3

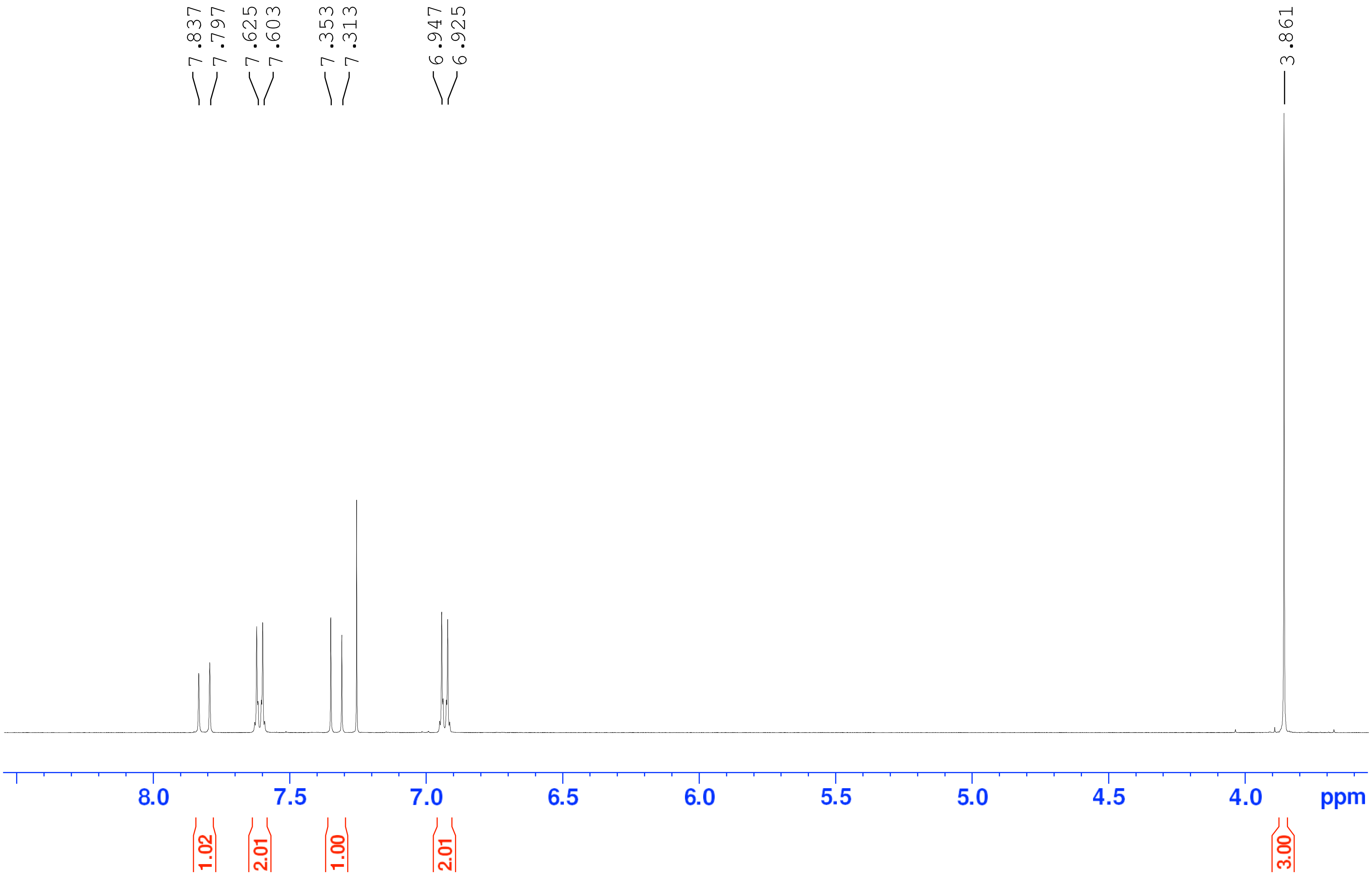

13C JMOD NMR (101 MHz)
Quaternary and CH2 up, CH3 and CH down
Compound 3c in CDCl3

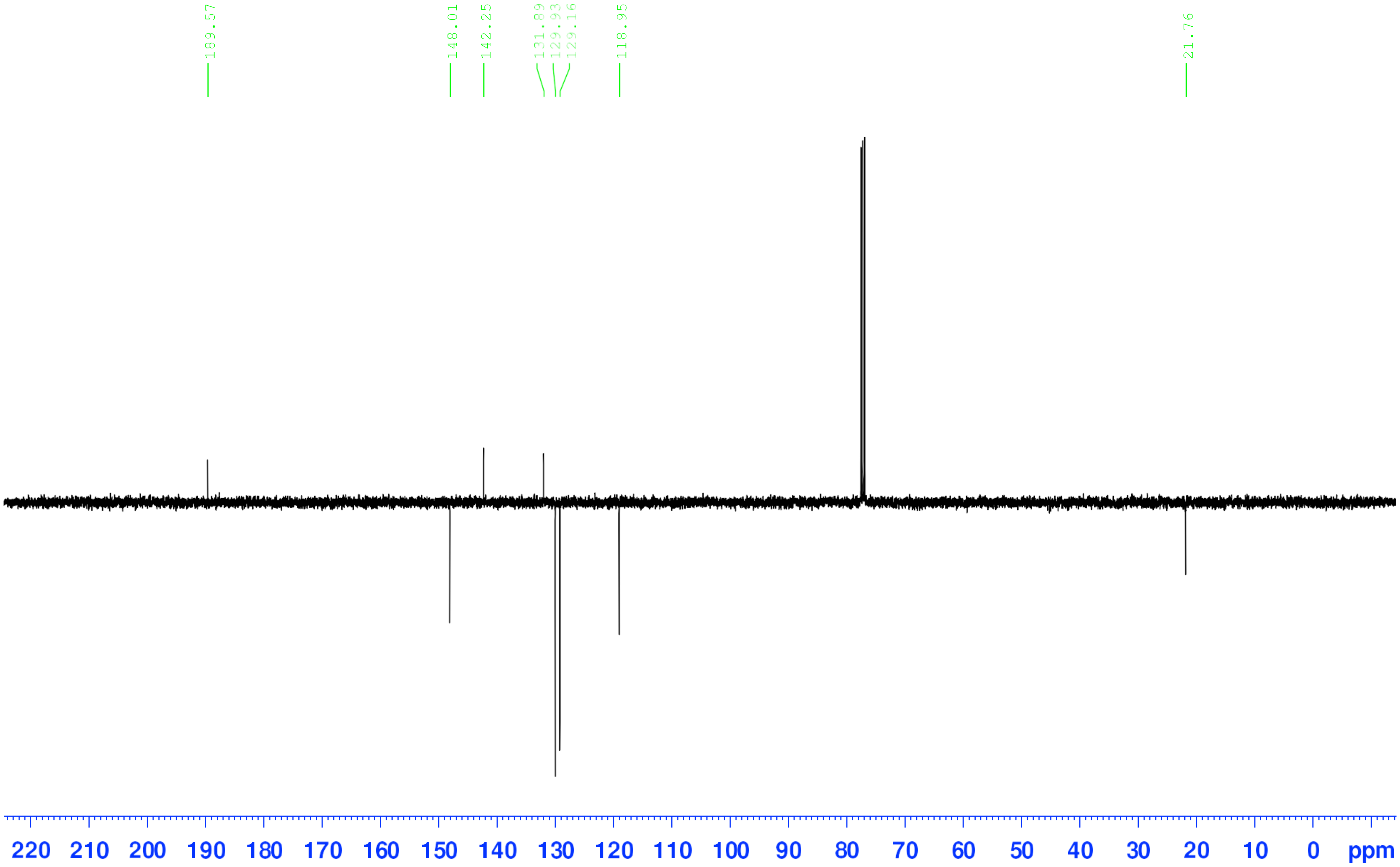

1H NMR (400MHz)
Compound 5a in DMSO-d6

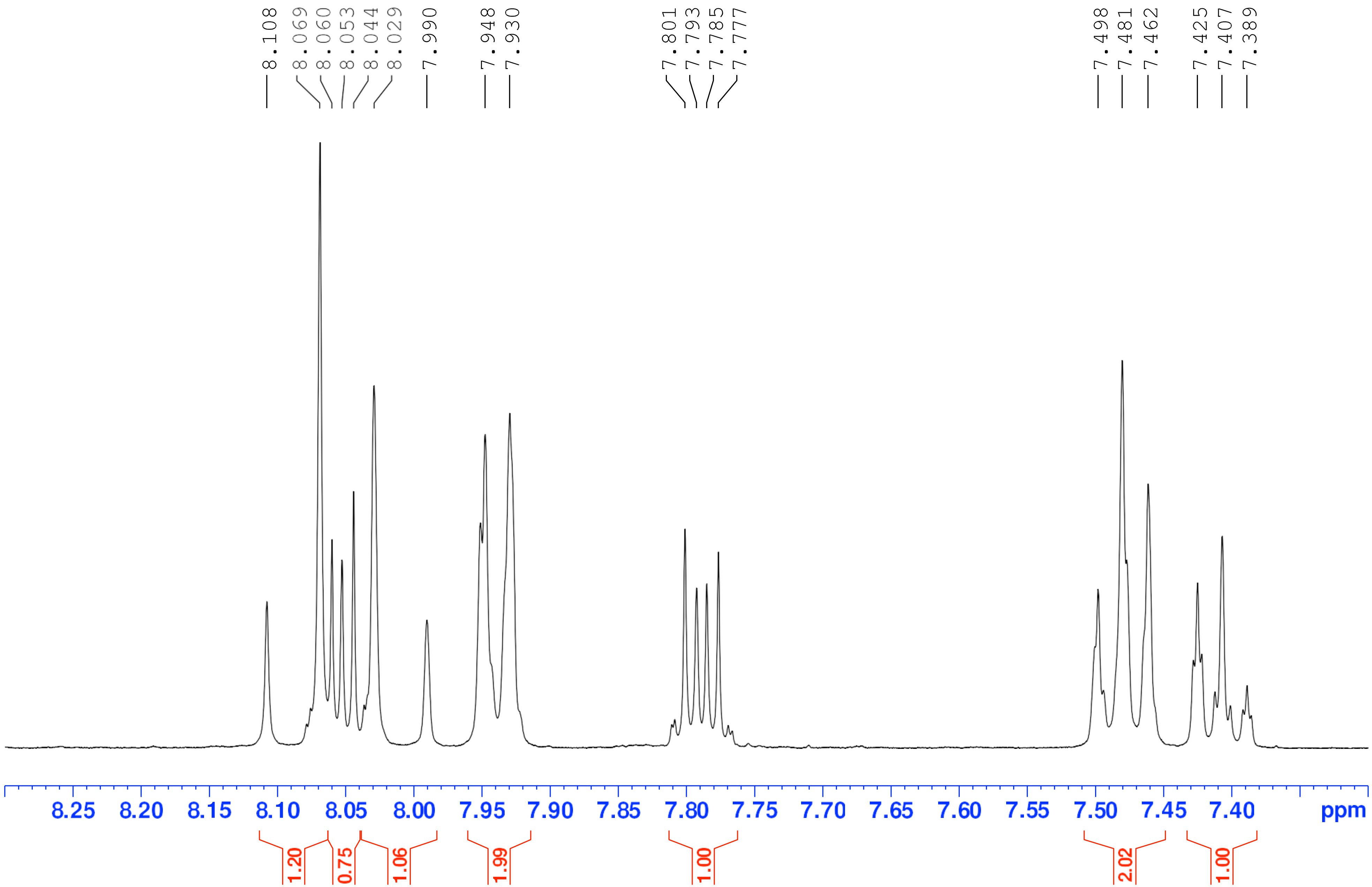

13C JMOD NMR (101 MHz)
Quaternary and CH2 up, CH3 and CH down
Compound 5a in DMSO-d6

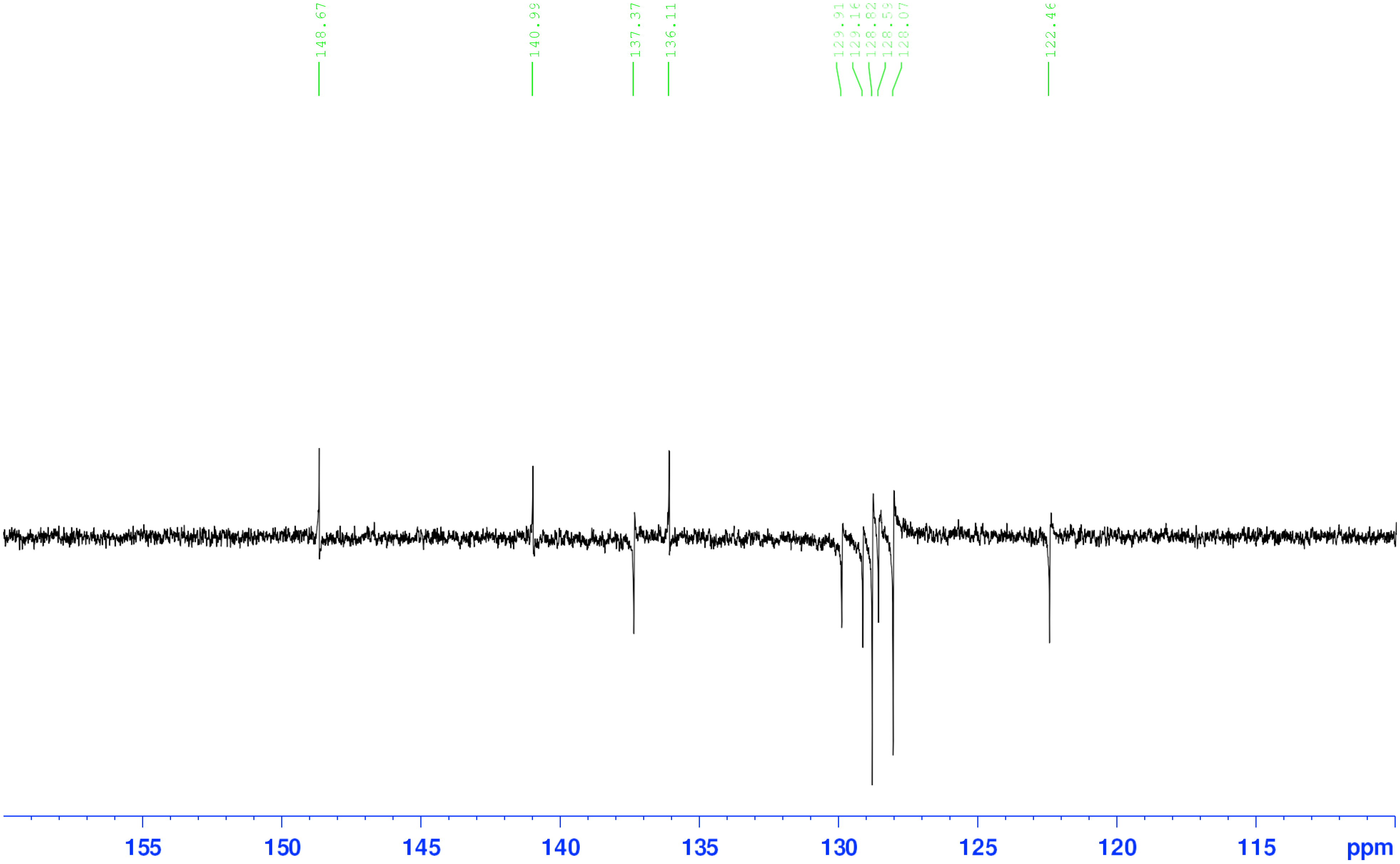

1H NMR (400 MHz)
Compound 5b in DMSO-d6

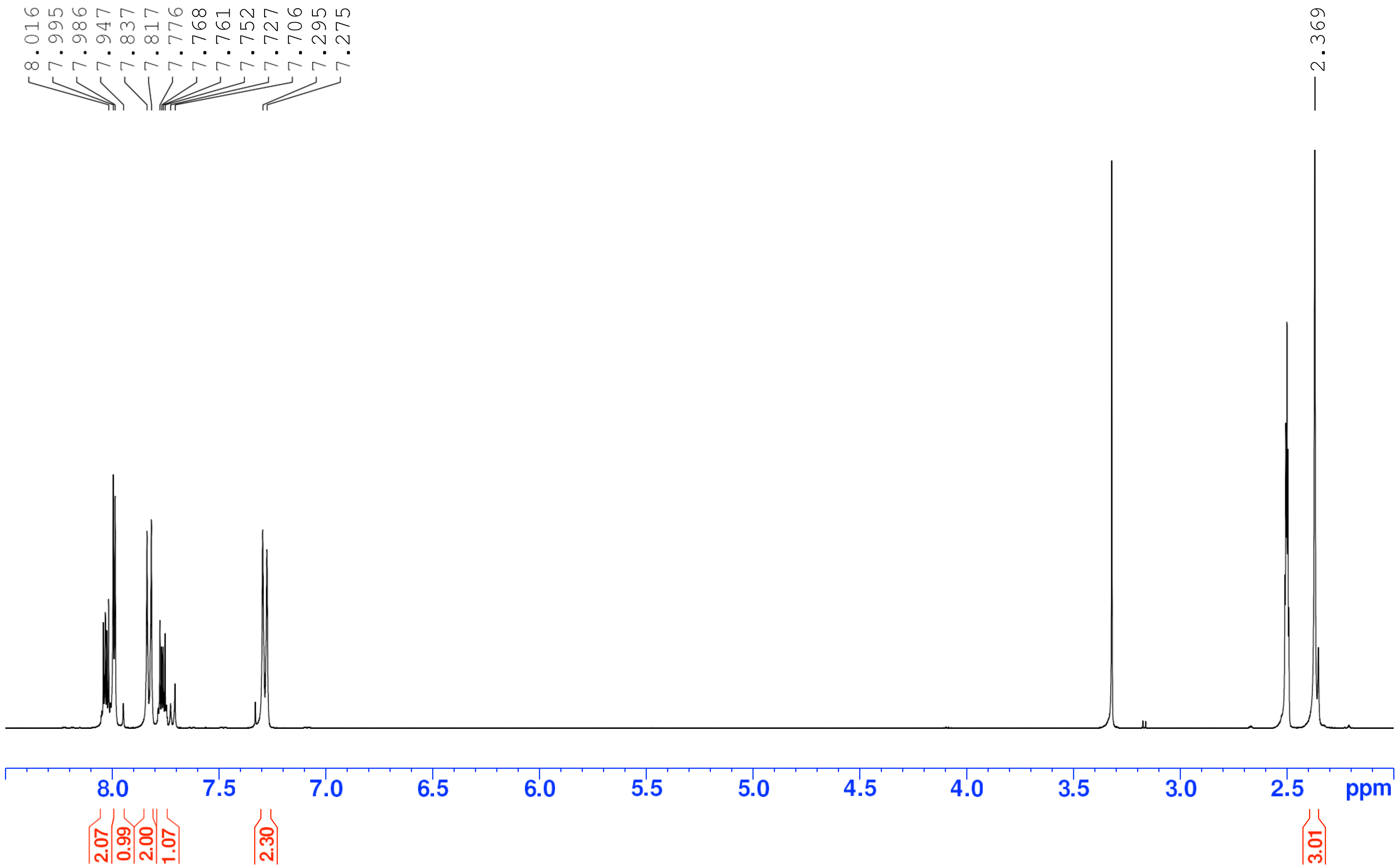

13C JMOD NMR (101 MHz)
Quaternary and CH2 up, CH3 and CH down
Compound 5b in DMSO-d6

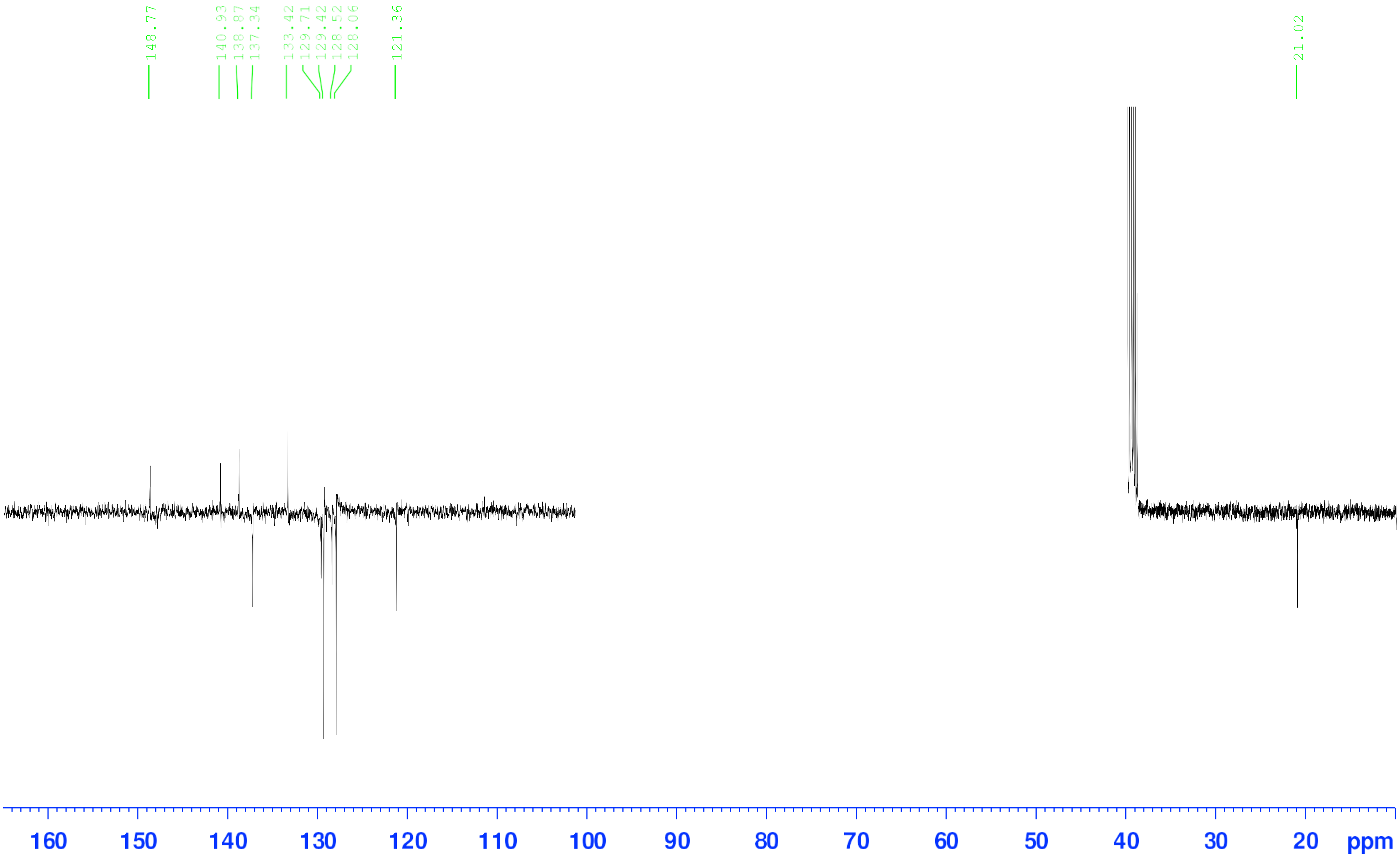

1H NMR (400 MHz)
Compound 5c in DMSO-d6

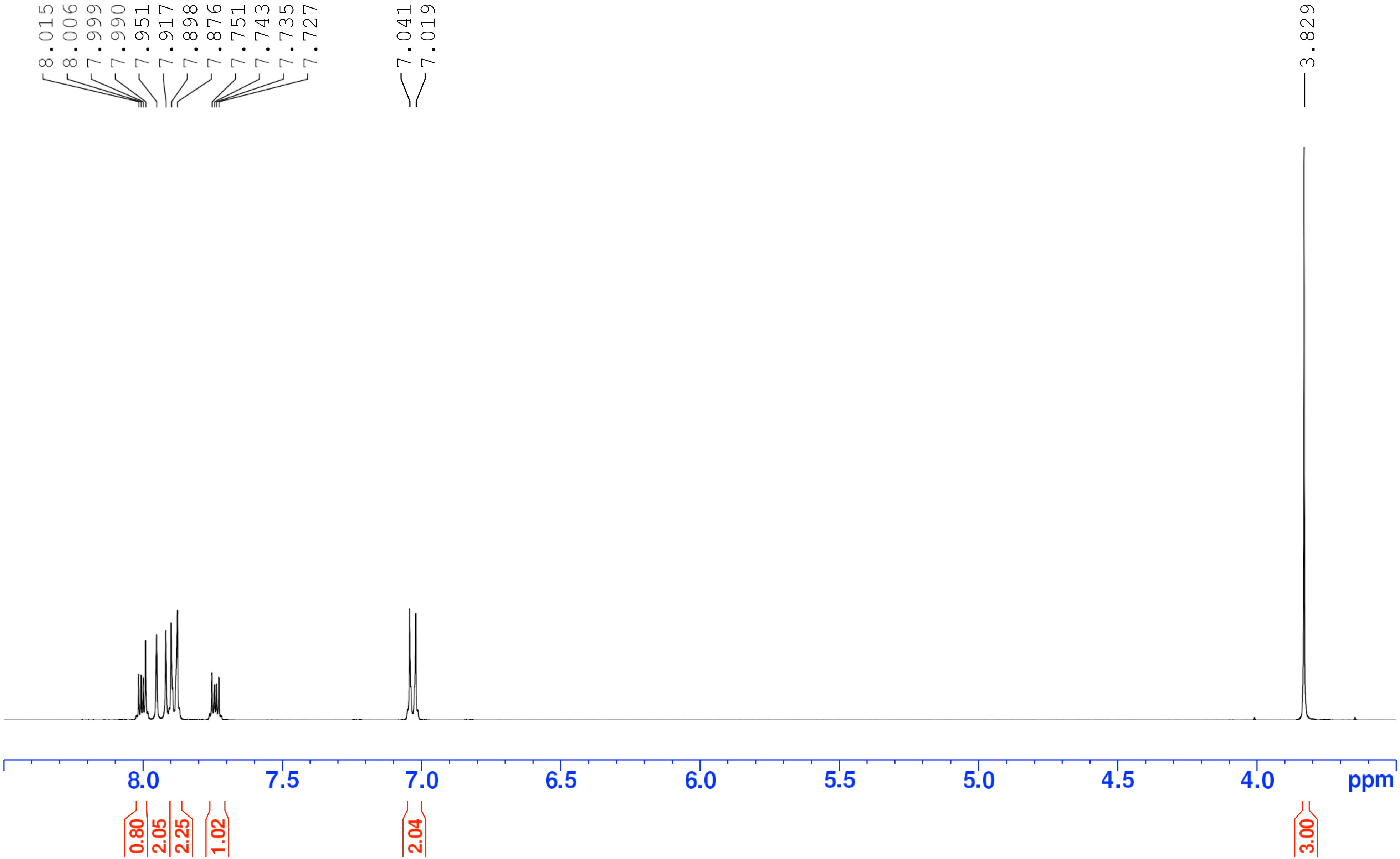

13C JMOD NMR (101 MHz)
Quaternary and CH2 up, CH3 and CH down
Compound 5c in DMSO-d6

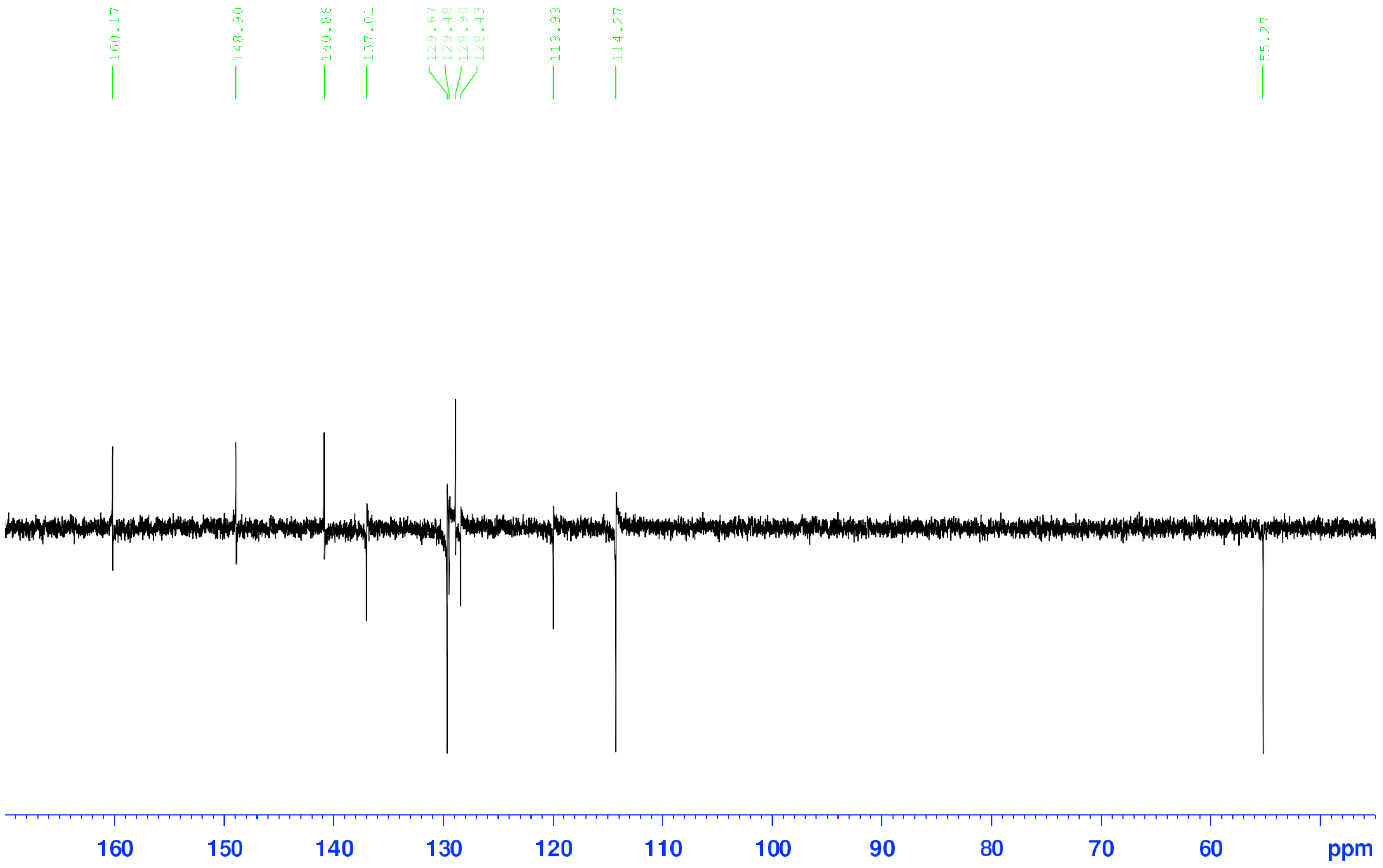

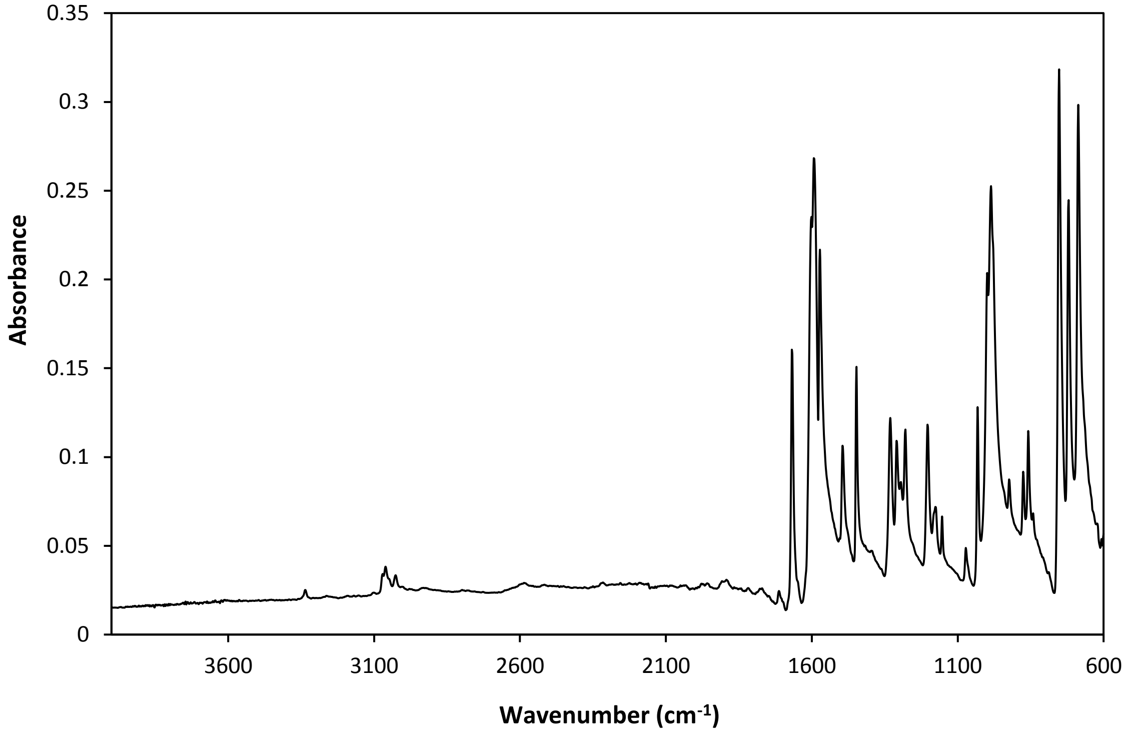

Figure S13. ATR-FTIR absorbance spectrum of cinnamil **3a**.

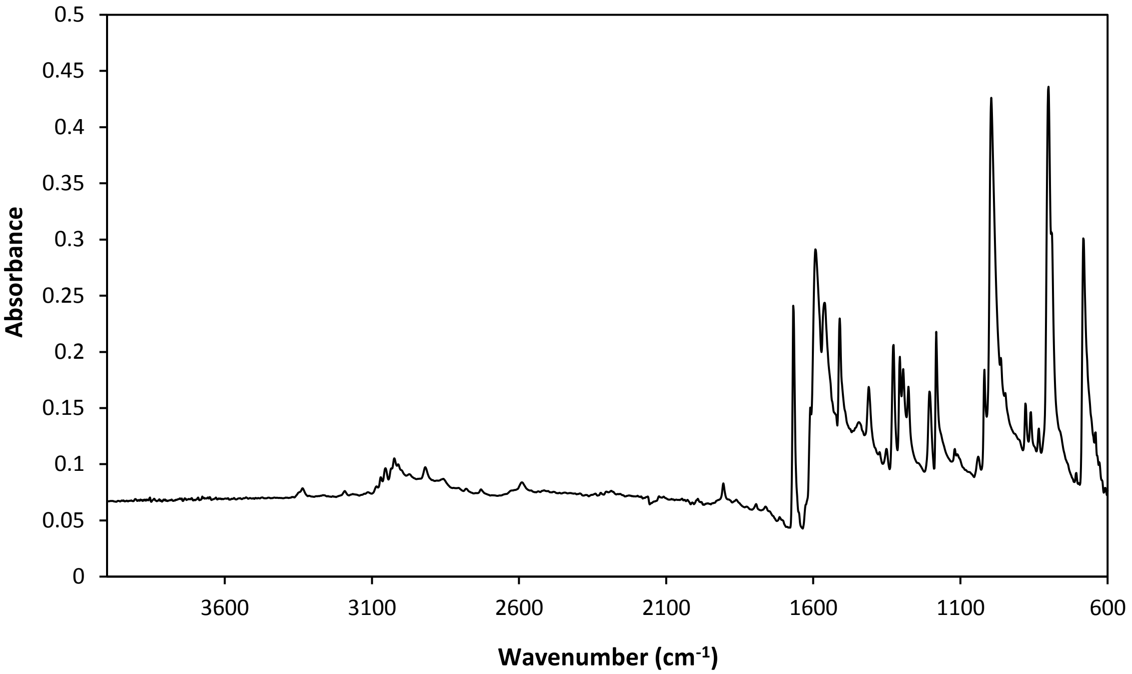

Figure S14. ATR-FTIR absorbance spectrum of cinnamil derivative **3b**.

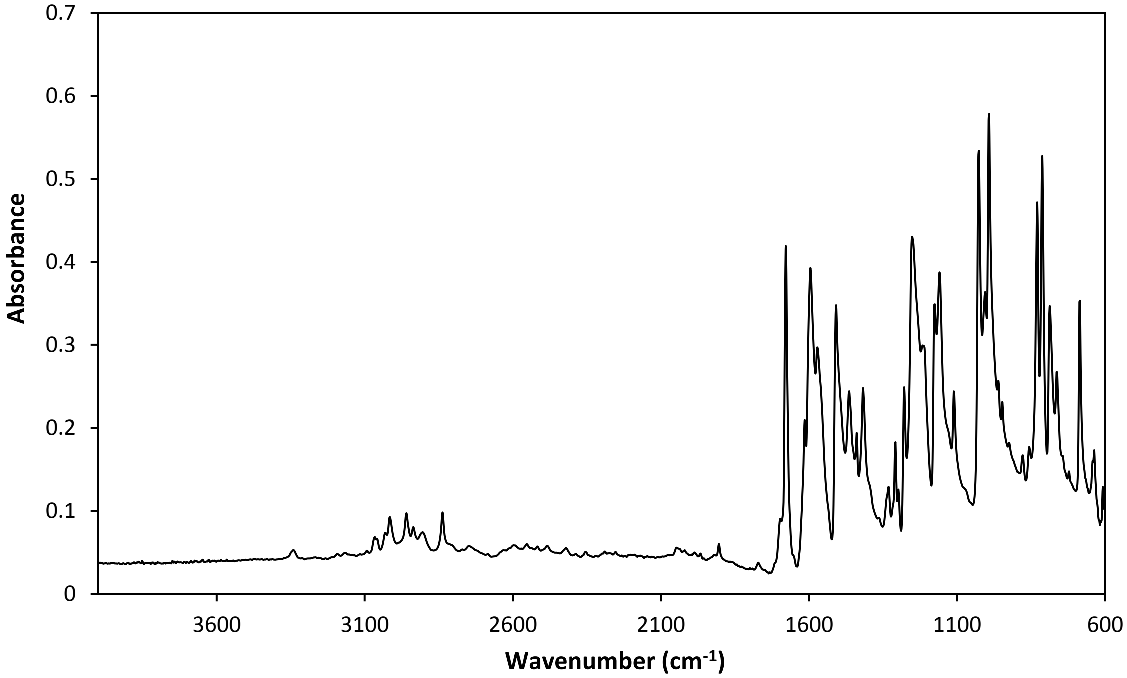

Figure S15. ATR-FTIR spectrum of cinnamil derivative **3c**.

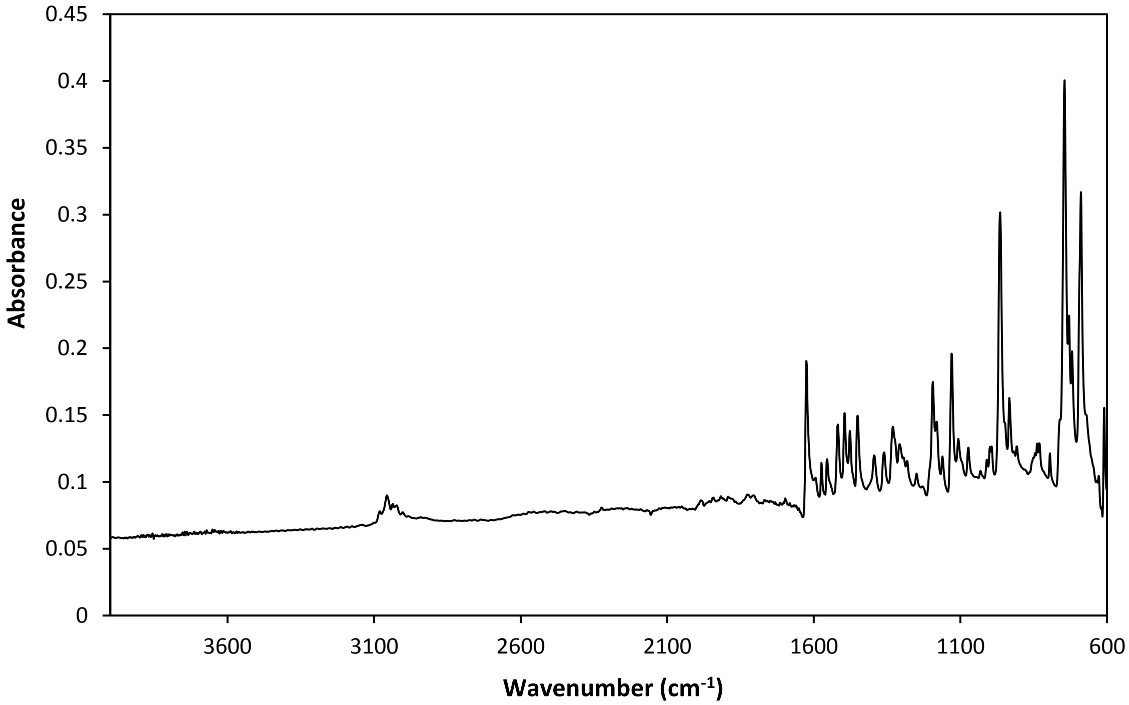

Figure S16. ATR-FTIR absorbance spectrum of quinoxaline derivative **5a**.

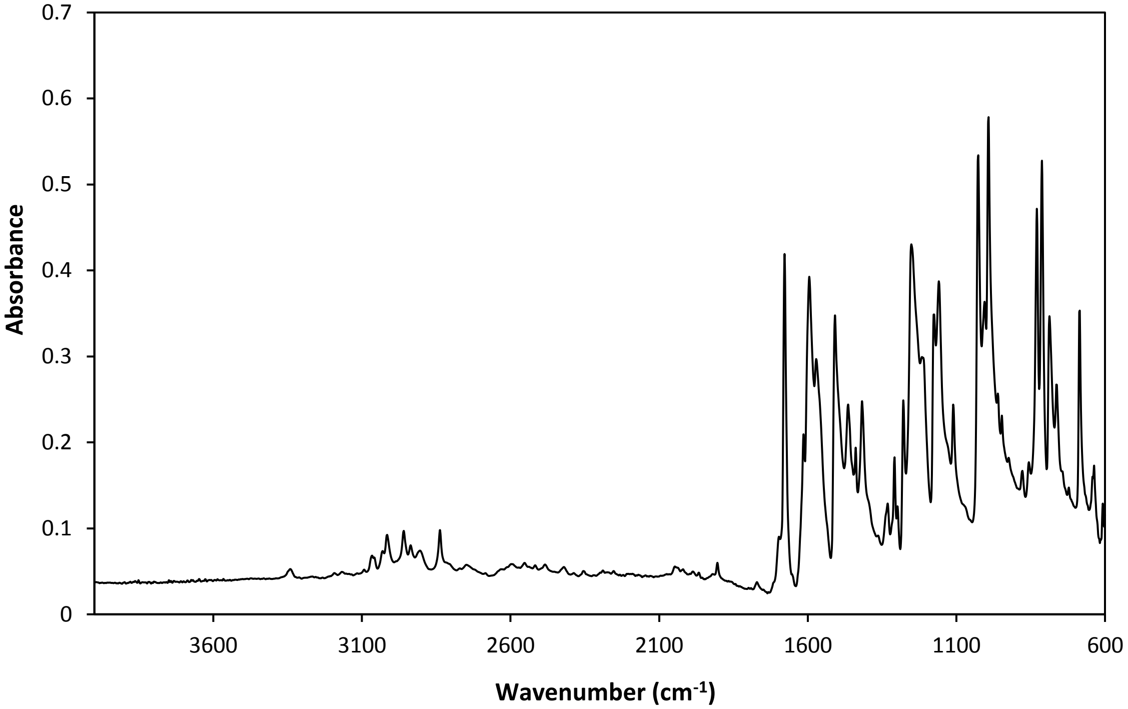

Figure S17. ATR-FTIR spectrum of quinoxaline derivative **5b**.

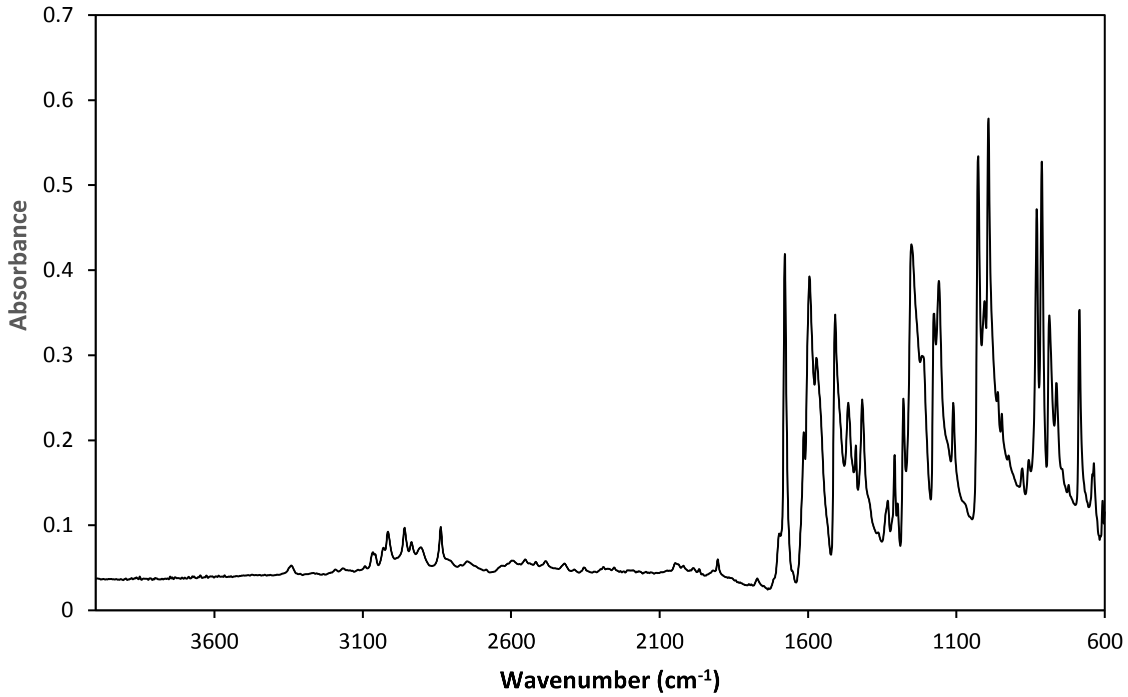

Figure S18. ATR-FTIR spectrum of quinoxaline derivative **5c**.

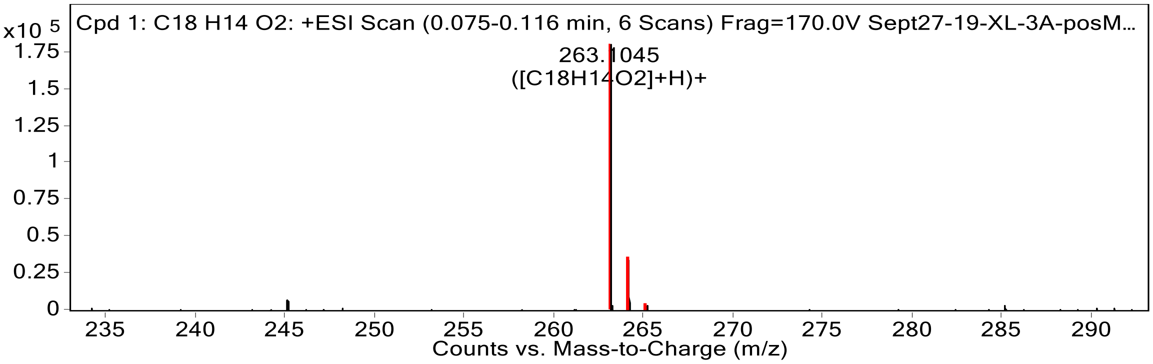

Figure S19. MS spectrum of cinnamil **3a**.

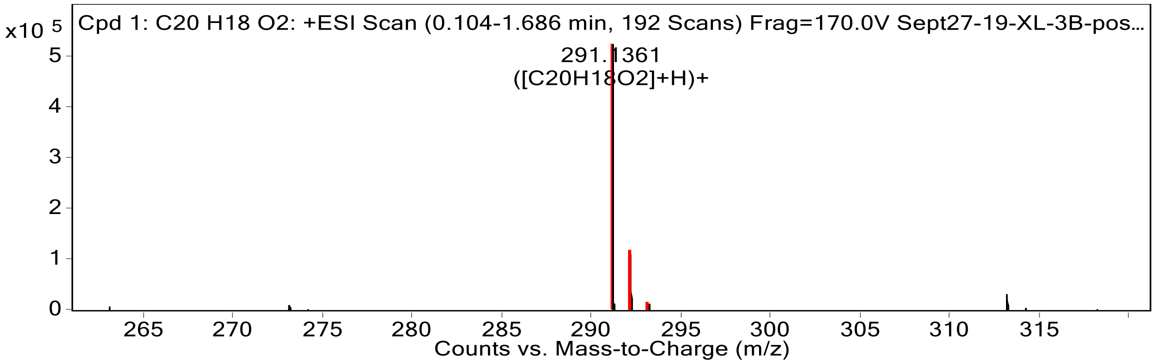

Figure S20. MS spectrum of cinnamil derivative **3b**.

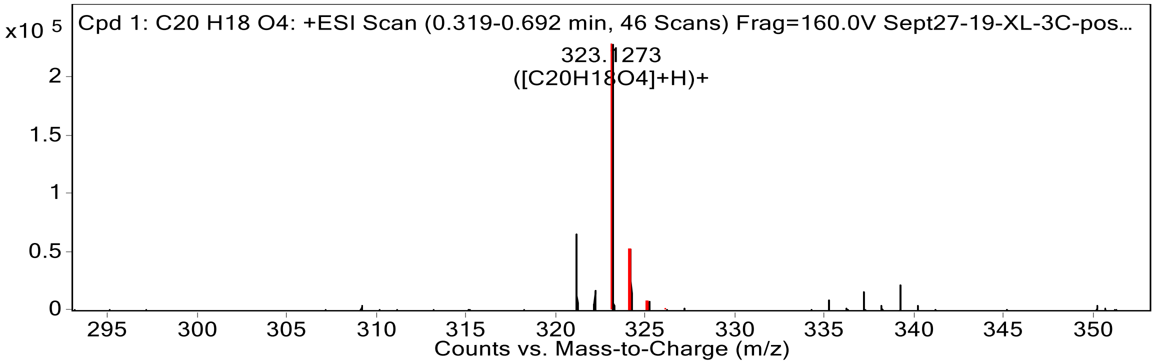

Figure S21. MS spectrum of cinnamil derivative **3c**.

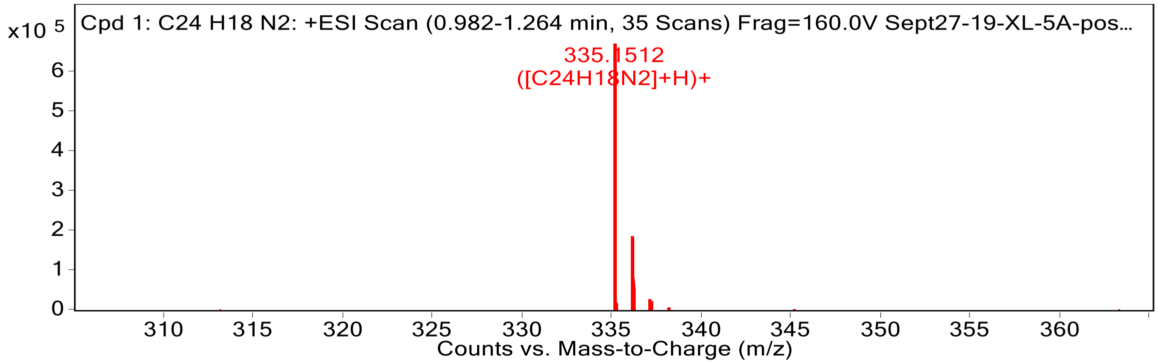

Figure S22. MS spectrum of quinoxaline derivative **5a**.

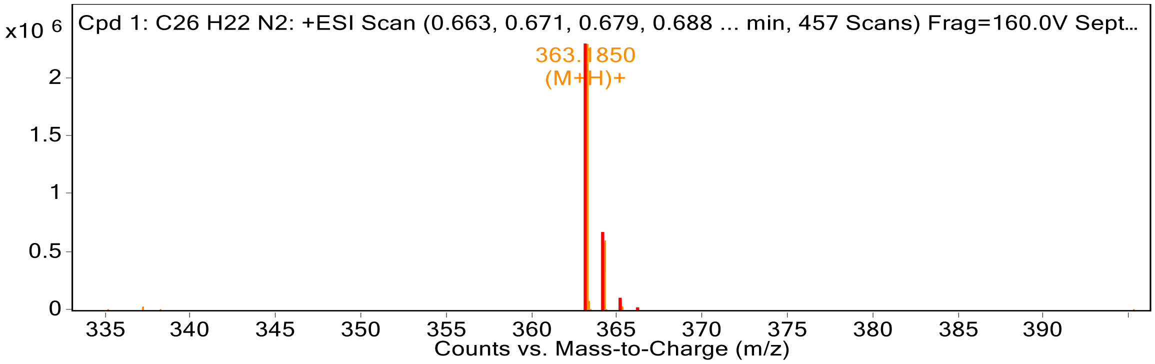

Figure S23. MS spectrum of quinoxaline derivative **5b**.

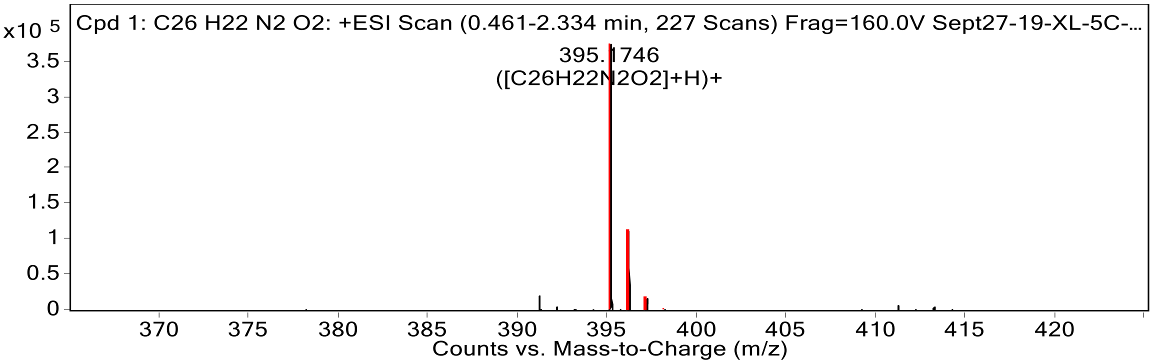

Figure S24. MS spectrum of quinoxaline derivative **5c**.

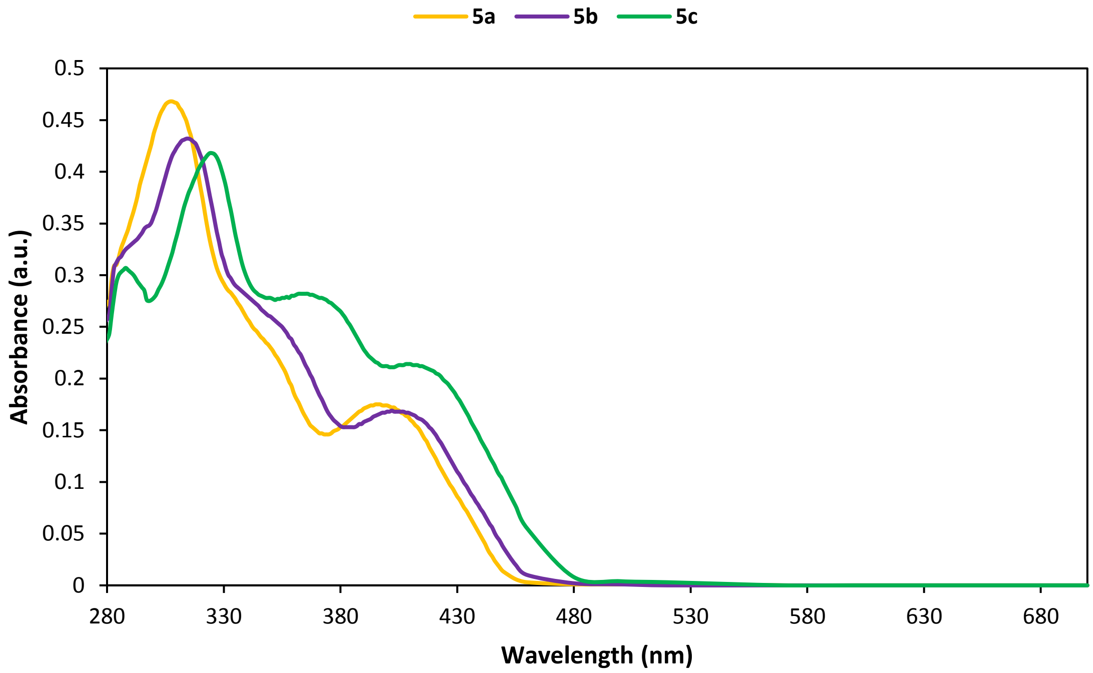

Figure S25. UV-Vis absorbance spectra of 10 μM acid-doped **5a-c** (1:5000 quinoxaline/acid molar ratio) in DMSO.
